# Little genetic differentiation as assessed by uniparental markers in the presence of substantial language variation in peoples of the Cross River region of Nigeria

**DOI:** 10.1186/1471-2148-10-92

**Published:** 2010-03-31

**Authors:** Krishna R Veeramah, Bruce A Connell, Naser Ansari Pour, Adam Powell, Christopher A Plaster, David Zeitlyn, Nancy R Mendell, Michael E Weale, Neil Bradman, Mark G Thomas

**Affiliations:** 1 Centre for Society and Genetics, University of California, Los Angeles, Rolfe Hall, Los Angeles, CA 90095-722, USA; 2 Novembre Laboratory, Department of Ecology and Evolutionary Biology, University of California, Los Angeles, 621 Charles E. Young Dr South, Los Angeles, CA 90095-1606, USA; 3 Centre for Research on Language Contact, Glendon College, York University, Toronto, Ontario M4N 3N6, Canada; 4 The Centre for Genetic Anthropology, University College London, Research Department of Genetics, Evolution and Environment, University College London, Gower Street, London WC1E 6BT, UK; 5 Molecular and Culture Evolution Laboratory, Research Department of Genetics, Evolution and Environment, University College London, Gower Street, London WC1E 6BT, UK; 6 Department of Anthropology, University of Kent, Canterbury CT2 7NR, UK; 7 Department of Applied Mathematics and Statistics, Stony Brook University, Stony Brook, NY 11794, USA; 8 Department of Medical and Molecular Genetics, King's College London, Guy's Tower, Guy's Hospital, London SE1 9RT, UK; 9 AHRC Centre for the Evolution of Cultural Diversity, Institute of Archaeology, University College London, London, WC1E 6BT, UK; 10 Deptartment of Evolutionary Biology, Evolutionary Biology Centre, Uppsala, University, Norbyvagen 18D, SE-752 36 Uppsala, Sweden

## Abstract

**Background:**

The Cross River region in Nigeria is an extremely diverse area linguistically with over 60 distinct languages still spoken today. It is also a region of great historical importance, being a) adjacent to the likely homeland from which Bantu-speaking people migrated across most of sub-Saharan Africa 3000-5000 years ago and b) the location of Calabar, one of the largest centres during the Atlantic slave trade. Over 1000 DNA samples from 24 clans representing speakers of the six most prominent languages in the region were collected and typed for Y-chromosome (SNPs and microsatellites) and mtDNA markers (Hypervariable Segment 1) in order to examine whether there has been substantial gene flow between groups speaking different languages in the region. In addition the Cross River region was analysed in the context of a larger geographical scale by comparison to bordering Igbo speaking groups as well as neighbouring Cameroon populations and more distant Ghanaian communities.

**Results:**

The Cross River region was shown to be extremely homogenous for both Y-chromosome and mtDNA markers with language spoken having no noticeable effect on the genetic structure of the region, consistent with estimates of inter-language gene flow of 10% per generation based on sociological data. However the groups in the region could clearly be differentiated from others in Cameroon and Ghana (and to a lesser extent Igbo populations). Significant correlations between genetic distance and both geographic and linguistic distance were observed at this larger scale.

**Conclusions:**

Previous studies have found significant correlations between genetic variation and language in Africa over large geographic distances, often across language families. However the broad sampling strategies of these datasets have limited their utility for understanding the relationship within language families. This is the first study to show that at very fine geographic/linguistic scales language differences can be maintained in the presence of substantial gene flow over an extended period of time and demonstrates the value of dense sampling strategies and having DNA of known and detailed provenance, a practice that is generally rare when investigating sub-Saharan African demographic processes using genetic data.

## Background

### The peoples and languages of the Cross River region

**Figure 1:**
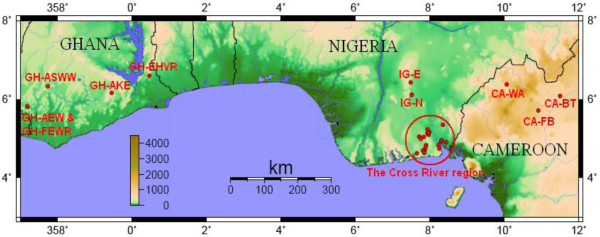
**Map showing where samples were collected**. Note:-Political borders are shown by black lines. Colour bar indicates elevation in metres.

The Cross River region (named after the river of the same name that passes through it) is situated in the extreme southeast of Nigeria, with its headwaters in the adjacent parts of Cameroon. The land to the north east of the Cross River region (Figure 1) is now generally accepted as the approximate location from which the expansion of the Bantu-speaking peoples began between three and five thousand years ago [1-3]. Bantu languages are now spoken throughout most of sub-Saharan Africa south of the equator. The Cross River region was also a major source of slaves during the Atlantic slave trade with Calabar, at the confluence of the Cross and Calabar Rivers, becoming both the region's principal urban centre and one of the trade's most active ports.

**Figure 2:**
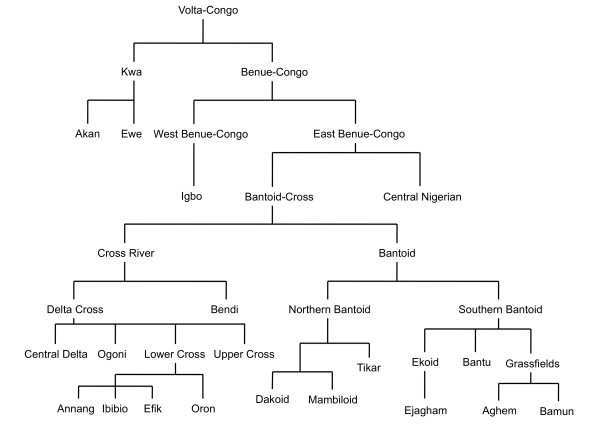
**Broad relationships of the differing language groups used or described in this work based on Williamson and Blench **[4]. Branch lengths are not informative.

Linguistically the Cross River region, for its size, is one of the most diverse in the world with more than 60 distinct languages still in daily use. Currently the accepted classification identifies 'Bantoid' and 'Cross River' as the two most important language groups found in the region (see Figure 2), though Williamson & Blench [4] argue that Cross River and Bantoid are sufficiently similar to be grouped together while still falling under Benue-Congo. The best studied subgroup within Cross River is Lower Cross, which is itself comprised of some twenty languages [5,6] including Anaang, Efik, Ibibio and Oron and is spoken over most of the lower region of the Cross River basin. Evidence from comparative linguistics, oral tradition [5,6] and documentary material [7,8] indicate that the Lower Cross languages together with the people that speak them are in the process of separating and spatially dispersing. Connell & Maison [6] suggest the major dispersal, with perhaps one or two earlier exceptions, began approximately 500-600 years ago and appears to have consisted of a general movement towards the coast from an inland-situated homeland, possibly due to pressure from incoming and expanding Igbo (some of the available oral traditions speak of these migrations and are examined in detail in Connell & Maison [6] and described briefly in the supplementary materials [Additional file 1: Supplemental Section 1]).

The primary branching of Bantoid is of North and South Bantoid. North Bantoid is comprised of Mambiloid, and more controversially Dakoid and Tikar (Boyd [9] questions the inclusion of Dakoid, while Connell [10] suggests the existence of the division itself is questionable). South Bantoid comprises numerous subgroups, including Bantu (itself made up of several hundred languages). Those in proximity to the Cross River region include Tivoid, Grassfields, Beboid, Nyang and importantly for this study, Ekoid, which contains Ejagham.

Another language grouping found partly in, but primarily to the west of the Cross River region, is Igboid, which consists mainly of a range of Igbo lects. Despite the geographical proximity of Igboland to the Cross River basin, Igboid languages are classified as West Benue-Congo [4], which reflects the considerable time (some thousands of years) since the existence of a common parent (viz. Proto Benue-Congo) of Igbo on the one hand and Cross River and Bantoid on the other (East Benue-Congo).

### Genetics and language

Comparative studies of differences among languages and uniparental genetic systems in populations have provided interesting insights into human history and social behaviour. Most studies have addressed relationships over a broad geographical canvas with considerable emphasis on the link between long-range language dispersals and the spread of agriculture [11-15]. More recent work has begun to examine, and find, relationships between linguistic and genetic variation at a finer scale (see for example the study of Lansing et al. [16] on the Sumba populations of eastern Indonesia). However such studies have yet to be applied to populations in sub-Saharan Africa.

Because of their location (situated in proximity to the probable Bantu homeland and an area that played a considerable role in the slave trade) and linguistic and cultural diversity, the peoples of the Cross River are of considerable interest to linguists (especially those concerned with historical linguistics and consequences of language contact), historians and other researchers interested in the mechanisms and implications of population movements. As variation in ethnic identities, cultural practices, oral histories and languages of the peoples of the Cross River are so well described with many tongues believed to have separated hundreds, and in some cases thousands, of years ago this region provides an excellent opportunity to examine possible associations of language and uniparental genetic differentiation on a fine scale.

### Aims of this study

In this study the Non-Recombining portion of the Y-chromosome (NRY) and mitochondrial DNA (mtDNA) in multiple well-characterised groups in the linguistically diverse Cross River region are analysed in one of the most densely sampled and well-defined human sub-Saharan African datasets collected to date from a localised geographic area. Groups speaking six different Benue-Congo languages that are well established in the Cross River region are included: Anaang, Efik, Ejagham, Igbo, Ibibio and Oron. DNA samples were collected from multiple locations and at various levels of ethnic identity (Table 1).

**Table 1:** Nigerian Cross River sample collection details.

***Code***	***Language***	***Place collected***	***Clan/Secondary affiliation***	***Latitude***	***Longitude***	***total n***
*SOUTH EAST NIGERIA*						

AN-EA	Annang	Afaha Esang, Ikot Ubom	Ediene Abak	5.050	7.717	26

AN-AO	Annang	Afaha Esang, Ikot Ubom	Afaha Obong	5.050	7.717	37

AN-IO	Annang	Abak, Ikot Obioma, Ikot Ekpene, Ukanafun		4.992	7.758	47

EF-EE	Efik	Eniong, Atan Ono Yom	Efut	5.167	7.983	50

EF-INE	Efik	Ikot Nakanda, Ikot Ene	Efut	4.908	8.442	48

EF-OEU	Efik	Oyo Efam, Ikot Abasi Obori	Uwanse	4.950	8.317	50

EK-CA	Ejagham	Calabar	Akampka	4.950	8.317	18

EK-CC	Ejagham	Calabar	Calabar	4.950	8.317	29

EK-CI	Ejagham	Calabar	Ikom	4.950	8.317	40

EK-NA	Ejagham	Netim	Akampka	5.350	8.350	51

IB-ANMWN	Ibibio	Afaha Nsit, Mbiokporo	Western Nsit	4.833	7.900	38

IB-EAEEUAE	Ibibio	Etebe Afaha Eket, Ekpene Ukpa	Afaha Eket	4.717	7.867	50

IB-EUE	Ibibio	Ette	Ukpom Ette	4.620	7.650	50

IB-IAAUA	Ibibio	Ikot Akpan, Afaha Ubiom	Awa	4.690	7.815	28

IB-IEINOI	Ibibio	Ikot Essien, Ikot Ntu	Oku-Iboku	5.133	7.933	50

IB-IMIEI	Ibibio	Ikot Mbonde, Ikot Ekang	Itam	5.042	7.842	50

IB-IOINO	Ibibio	Ikot Oku, Ikot Ntuenoku	Oku	5.100	7.967	50

IB-MNENN	Ibibio	Mkpok Ndon Eyo	Nnung Ndem	4.633	7.850	50

IB-NEI	Ibibio	Ndiya	Edienne Ikono	4.783	7.883	50

IB-OII	Ibibio	Obong Itam	Itam	5.133	7.967	50

IB-ONMNI	Ibibio	Onoh, Ntan Mbat	Ntan Ibiono	5.233	7.933	50

IG-C	Igbo	Calabar		4.950	8.317	100

OR-AO	Oron	Oron	Afaha Okpo	4.833	8.233	28

OR-ENEEAU	Oron	Eyo Nsik, Eyo Ekpe	Afaha Ukwong	4.750	8.250	73

						

IG-E	Igbo	Enugu		6.433	7.483	57

IG-N	Igbo	Nenwe		6.117	7.517	52

						

*CAMEROON*						

CA-BT	Tikar	Bankim		6.083	11.500	34

CA-FB	Bamun	Foumban		5.717	10.917	117

CA-WA	Aghem	Wum		6.383	10.067	118

						

*GHANA*						

GH-AEW	Akan	Enchi		5.817	-2.817	21

GH-AKE	Akan	Kibi		6.167	-0.550	51

GH-ASWW	Akan	Sefwi-Wiawso		6.333	-2.267	22

GH-FEWR	Akan	Enchi		5.817	-2.817	61

GH-EHVR	Ewe	Ho		6.600	0.467	88

The principal aim of this study was to establish whether or not there has been substantial inter-language group gene flow in the Cross River region. A crude expectation of just over 10% for the level of gene flow per generation between different language groups (regardless of sex) can be generated based on the whether the parents of individuals collected for this study spoke the same primary language [Additional File 2: Supplemental Table S1]. While in a sociological-anthropological context it may appear that language is a strong factor in mate choice, under a simple Wright island model, with 'islands' of at least 1000 individuals, we expect a Fixation Index of at most 0.002 with this migration rate, a very low value that indicates a substantial amount of gene flow between 'islands' [17]. However it should be noted that the sociological information on inter-group gene flow is based on data from only the last two generations before present and this high value of 10% may be only be a recent phenomenon and have very little effect on genetic structure.

African genetic diversity and its positive correlation with both geography and language has previously been well described at the continent-wide scale for both uniparental and autosomal markers [14,18,19]. However attempts to investigate the relationship at finer scales, for example within language families, have demonstrated this relationship breaking down on occasion. Whether this is a real and widespread phenomenon or simply a result of the unsuitability of the datasets utilised with regard to sampling density is unclear. Having a good understanding of the relationship between geographic/linguistic scale and human genetic variation is important from linguistic, anthropological and medical perspectives. Therefore, in order to compliment existing studies conducted at very broad scales we also examined the Cross River region within the somewhat intermediate geographical context of West Central Africa by analysing additional groups resident in the neighbouring Northwest Province (NWP) of Cameroon and more distant Ghanaian populations (see Figure 1 and Table 1). Gene flow between these three regions is likely to be low given the large distances involved and therefore observable differences among the NRY and mtDNA profiles of these three regions would be expected in comparison to the Cross River scale. Finally this study will also provide vital additional information on the overall pattern of genetic variation in sub-Saharan Africa such as the distribution of the widespread Y-haplogroup E1b1a and its subclades.

## Results

### Investigating potential language structuring in the Cross River region

Using pooled datasets of speakers of the six different linguistic groups sampled in the Cross River region (where clan/secondary affiliations were ignored) the hierarchical Analysis of Molecular Variance (AMOVA)-based Fixation indexes were not significant at any NRY [Additional file 2: Supplemental Table S2] or mtDNA [Additional file 2: Supplemental Table S3] level (P > 0.100) (see Table 2 for all AMOVA results). However to take into account any differences between language groups due to differences within language groups each clan was analysed separately but within a framework where they were hierarchically grouped by their language spoken. Again the AMOVA-based Fixation Indices for among-language-group differences were not significant at any NRY or mtDNA level of analysis (P-value > 0.105).

**Table 2:** Hierarchical AMOVA results of Cross River, Cameroonian and Ghanaian groups at various molecular levels.

	***Cross River region (n = 24)***		***Cameroonian NWP (n = 3)***		***Ghana (n = 5)***		***Ibibio (n = 11)***		***Cross River pooled groups of language speakers (n = 6)***		***Cross River clans grouped by language (n = 6,24)***		***Cross River clans grouped by language with 2 Igbo populations (n = 6,26)***		***Cross River region + Ghana+ Cameroonian NWP (n = 3,32)***	
***Genetic system and level of molecular resolution***	***F_ST_***	***P-value***	***F_ST_***	***P-value***	***F_ST_***	***P-value***	***F_ST_***	***P-value***	***F_ST_***	***P-value***	***F_CT_***	***P-value***	***F_CT_***	***P-value***	***F_CT_***	***P-value***

*NRY UEP F_ST_*	0.002	**0.330**	0.109	**< 0.001#**	0.023	0.024*	0.003	**0.301**	-0.002	**0.737**	-0.003	**0.810**	0.001	**0.339**	0.033	**0.002$**

*NRY UEP+MS F_ST_*	-0.001	**0.664**	0.071	**< 0.001#**	0.003	**0.181**	-0.002	**0.891**	0.000	**0.450**	0.000	**0.340**	0.000	**0.296**	0.015	**0.001#**

*NRY MS R_ST_*	0.004	**0.132**	0.139	**<0.001#**	0.008	**0.167**	0.004	**0.180**	-0.001	**0.603**	-0.003	**0.888**	-0.002	**0.774**	0.025	0.025*

*mtDNA HVS-1 VSO F_ST_*	0.000	**0.242**	0.010	**<0.001#**	0.000	**0.374**	0.001	**0.138**	0.001	**0.100**	0.001	**0.130**	0.000	**0.202**	0.005	**<0.001#**

*mtDNA HVS-1 K2*	-0.001	**0.663**	0.001	**0.351**	0.001	**0.368**	0.000	**0.498**	0.001	**0.191**	0.002	**0.105**	0.002	**0.086**	0.016	**<0.001#**

Symbol following value indicates significance level of Fixation Indices P-values: * = 0.05 < P < 0.01, $ = 0.01 < 0.001, # = P < 0.001. Each grouping is followed, indicated by 'n', by the number of groups and, if applicable, the number of individual populations analysed.

Though the Fixation Indices discussed above indicate a lack of among-group structure a small number of significant individual pairwise differences were observed at every NRY and mtDNA level (0-1.4% of pairwise comparisons for a particular level of NRY or mtDNA analysis were significant at least at the 1% level, within the expected Type 1 error range [Additional file 2: Supplemental Table S4] [Additional file 2: Supplemental Table S5])

We conducted simulations [Additional file 1: Supplemental Section 2] replicating NRY UEP haplogroup and six microsatellite (UEP+MS) haplotype and mtDNA Hypervariable Segment -1(HVS-1) haplotype population dynamics in the Cross River region under realistic demographic parameters. The number of significant (P < 0.05) population pairwise genetic distances observed (5-6% of all pairwise comparisons) was much less than expected even for migration rates as high as 0.3 (23% of all pairwise comparisons) using simulated data. In addition the simulations showed that at such high migration rates the simulated AMOVA-based Fixation Indices were still not as low as for our observed data and that most population pairwise significant differences were stochastic (possibly driven by random sampling effects) and of a transient nature (persisting for an average of two generations before the general high migration rate of the world "re-homogenised" the populations). Thus the results of our simulations are compatible with the scenario of the Cross River region being a homogenous system with high inter-group migration.

### Cross River region and Igboland

Calabar is considered a particularly cosmopolitan city where different ethnicities reside together at an unusually high frequency for the Cross River region as a whole. Therefore two groups from Igboland to the west of the Cross River region (IG-E and IG-N) were added to the inter-language group analysis to take into account the potentially unusually high levels of inter-ethnic admixture that may have taken place involving Igbo from Calabar. The AMOVA-based F_CT _(the among-group Fixation Index) values (see Table 2) were not noticeably different at any NRY or mtDNA levels when the IG-N and IG-E were grouped with the Igbo-speaking group from Calabar (all other language group structures were the same) and none of the F_CT _values were significant (P-value > 0.086). However there was a notable and substantial increase in the number of pairwise significant differences involving the two Igboland groups and other Cross River clans [Additional file 2: Supplemental Table S4] [Additional file 2: Supplemental Table S5], especially for IG-N at the UEP and UEP+MS levels where 22/24 comparisons were significantly different at the 5% level (15-16 at the 1% level).

### The Cross River region within the context of West Central Africa

Using three pooled datasets consisting of the 24 Cross River region clans, five Ghanaian groups and three Cameroonian NWP groups (note that the Tikar population, CA-BT, strictly lie in the Adamaoua Province close to the NWP border) respectively, pairwise ETPD showed significant differences at the 1% threshold between all three datasets at all NRY and mtDNA levels while NRY R_ST _and mtDNA K2 (see Methods section for explanation of K2) genetic distances were also significant at the 1% threshold [Additional File 2: Supplemental Table S6]. Once again, to account for possible within-region differentiation the Cross River clans and Ghanaian and Cameroonian groups were analysed within a framework where populations were also hierarchically grouped by their country of origin. The AMOVA-based Fixation Indices for among-country-group differences were significant at the 5% threshold using R_ST _and were significant at the 1% level using UEP defined haplogroups and UEP+MS haplotypes and at both levels of mtDNA analysis.

The Cameroonian NWP populations tended to demonstrate more pairwise significant differences (both in number and significance level) than Ghanaian populations when compared to Cross River clans [Additional file 1: Supplemental Figure S1] [Additional file 2: Supplemental Table S4] [Additional file 2: Supplemental Table S5]. Pairwise comparisons via genetic distances and ETPD [Additional file 2: Supplemental Table S4] [Additional file 2: Supplemental Table S5] also show that at the UEP+MS, R_ST _and mtDNA haplotype levels (and to some extent mtDNA K2 levels) pairwise comparisons between Ghanaian and Cameroonian populations were highly significant. It was noticeable that the AMOVA-based Fixation index for the Cameroonian NWP alone was highly significant at all levels (P < 0.001) except based on mtDNA K2 distances, while Ghana was more homogenous, only showing significance at 5% at the UEP level (see Table 2).

**Figure 3:**
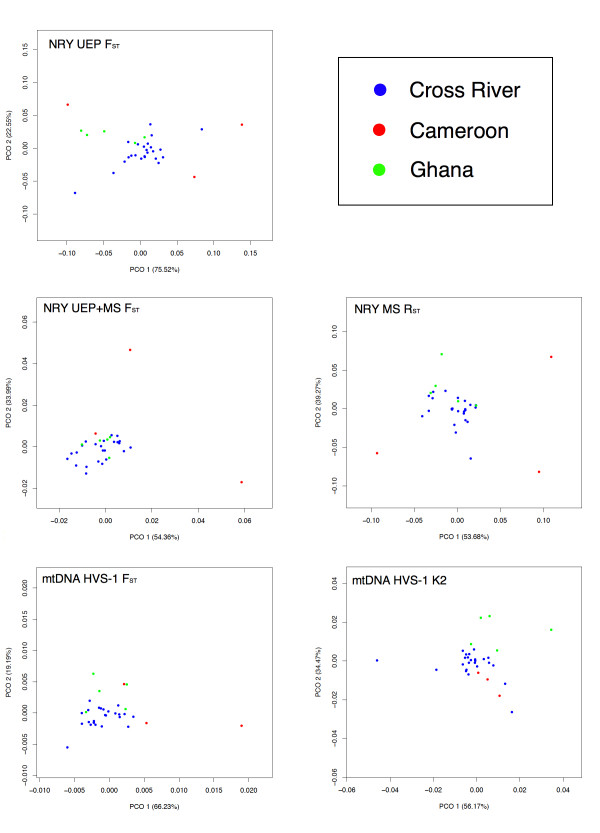
**Various PCO plots at different NRY and mtDNA analysis levels for populations from the Cross River region, the Cameroonian NWP and Ghana**.

Principle Co-Ordinate (PCO) plots of NRY and mtDNA genetic distances at various levels of resolution showed a general pattern (see Figure 3) at all levels where the Cross River populations clustered together, with the Cameroonian and Ghanaian populations tending to lie on the periphery of this cluster and Cameroonian populations being noticeably more disparate than the more homogenous Ghanaian populations.

### Are there correlations of genetic distances and geographic and linguistic distances?

A Mantel test of correlation between genetic and linguistic distance for the Cross River clans showed no correlation at any NRY or mtDNA level (P > 0.271) (see Table 3 for all Mantel and Partial Mantel test results) apart from at the UEP+MS level (P = 0.036). This correlation, albeit only moderately significant, was maintained even when the comparison was controlled for geographic distance (r = 0.333, P = 0.028). No correlation was found between genetic and geographic distance at any level, even when holding linguistic distance constant (P > 0.359). Consistent with the increased number of significant pairwise differences described earlier, expanding the Cross River dataset to include the Igboland populations did reveal significant correlations between both NRY UEP and UEP+MS F_ST_s and both geographic and linguistic distance.

**Table 3:** Results of Mantel and Partial Mantel tests at different levels of NRY and mtDNA analysis using various distance matrices.

			***Genetic distance matrix type calculated***									
			
			***NRY UEP-based F_ST_***		***NRY UEP+ms-based F_ST_***		***NRY Microsatellite-based R_ST_***		***mtDNA VSO-based F_ST_***		***mtDNA VSO-based K2***	
***Correlation Analysis type***		***Groups utilised***	***R***	***P-value***	***R***	***P-value***	***R***	***P-value***	***R***	***P-value***	***R***	***P-value***

*Mantel*		*Cross River + Cameroon + Ghana*	*0.382*	**0.004$**	*0.182*	**0.141**	*0.235*	**0.055**	*0.300*	0.037*	*0.432*	**<0.001#**
		
	*Geography*	*Nigeria (Includes IG-N and IG-E)*	*0.450*	**0.005$**	*0.377*	0.024*	*0.079*	**0.258**	*0.098*	**0.214**	*0.142*	**0.175**
		
		*Cross River*	*0.022*	**0.360**	*-0.049*	**0.688**	*0.008*	**0.460**	*-0.033*	**0.659**	*0.018*	**0.378**
	
		*Cross River + Cameroon + Ghana*	*0.341*	**0.001#**	*0.391*	**<0.001#**	*0.317*	**0.002$**	*0.372*	**<0.001#**	*0.347*	**<0.001#**
		
	*Linguistics*	*Nigeria (Includes IG-N and IG-E)*	*0.217*	0.049*	*0.414*	**0.001$**	*0.111*	**0.160**	*0.113*	**0.146**	*0.080*	**0.260**
		
		*Cross River*	*-0.014*	**0.499**	*0.305*	0.036*	*0.067*	**0.293**	*0.074*	**0.271**	*-0.015*	**0.513**

*Partial Mantel*		*Cross River + Cameroon + Ghana*	*0.235*	0.036*	*-0.073*	**0.6736**	*0.057*	**0.318**	*0.103*	**0.191**	*0.298*	**0.008$**
		
	*Geography controlling Linguistics*	*Nigeria (Includes IG-N and IG-E)*	*0.404*	**0.007$**	*0.225*	**0.090**	*0.029*	**0.403**	*0.051*	**0.343**	*0.119*	**0.197**
		
		*Cross River*	*0.028*	**0.359**	*-0.148*	**0.962**	*-0.011*	**0.549**	*-0.056*	**0.753**	*0.023*	**0.379**
	
		*Cross River + Cameroon + Ghana*	*0.149*	**0.088**	*0.358*	**0.003$**	*0.227*	0.028*	*0.251*	0.015*	*0.120*	**0.121**
		
	*Linguistics controlling Geography*	*Nigeria (Includes IG-N and IG-E)*	*0.004*	**0.464**	*0.288*	0.023*	*0.084*	**0.225**	*0.076*	**0.236**	*0.014*	**0.438**
		
		*Cross River*	*-0.021*	**0.500**	*0.333*	0.028*	*0.067*	**0.293**	*0.087*	**0.233**	*-0.021*	**0.523**

Significance code is the same as Table 2.

When the 24 Cross River region populations were considered with the five Ghanaian and three Cameroonian groups highly significant correlations were found between genetic and linguistic distance (P < 0.01) at all NRY and mtDNA levels. Highly significant correlations were also found between genetic and geographic distance at the UEP and mtDNA K2 levels (P < 0.01) while using the mtDNA F_ST _distance produced a significant correlation at 5% significance (P = 0.037). When a partial Mantel test was applied a contrasting pattern was observed such that the correlation with linguistic distance was maintained at the UEP+MS, MS and mtDNA F_ST _levels while the evolutionarily deeper UEP and mtDNA K2 distances showed correlations with geographic distance, though all P-values were noticeably increased.

### NRY Haplogroup distribution

Ten haplogroups were observed in the Cross River dataset (n = 1081) (See Table 4). The overall modal haplogroup was E1b1a7 (45%) closely followed by E1b1a8 (38%) (see Table 2). In the majority of clans (17/24) the E1b1a7 haplogroup was modal (mean: 0.46, variance: 0.006, range: 0.30-0.67). A median-joining network constructed using all non-singleton NRY microsatellite haplotypes [Additional file 1: Supplemental Figure S2] displayed two striking features. Firstly BR*(xDE, JR) haplotypes appeared in two distinct clusters. Given the particularly crude assignment of NRY to this haplogroup, which encompasses a number of prominent subclades, it is likely that at least one of these represent the sub-Saharan African-specific Haplogroup B, while the other cluster may contain a typically non-sub-Saharan African haplogroup (for example Haplogroups F, G and I have been found at low frequencies amongst typically African ethnic groups in the Democratic Republic of São Tomé and Príncipe [20], presumably because of European (especially Portuguese) introgression during the Slave trade.

**Table 4:** NRY Haplogroup proportions in Cross River, Cameroonian NWP and Ghanaian groups.

NRY UEP Haplogroup	P*(xR1a)	BR*(xDE, JR)	E*(xE1b1a)	K*(xL, N1c, O2b, P)	Y*(xBR, A3b2)	DE*(xE)	A3b2	J	E1b1a*	E1b1a7	E1b1a8
*AN-AO*	0.00	0.03	0.00	0.00	0.00	0.00	0.00	0.00	0.07	0.30	**0.60**

*AN-EA*	0.00	0.08	0.08	0.00	0.00	0.00	0.00	0.00	0.04	**0.42**	0.38

*AN-IO*	0.00	0.17	0.02	0.00	0.00	0.00	0.00	0.00	0.00	**0.43**	0.38

*EF-EE*	0.00	0.06	0.04	0.00	0.00	0.00	0.00	0.00	0.02	**0.45**	0.43

*EF-INE*	0.02	0.19	0.02	0.00	0.00	0.00	0.00	0.00	0.13	**0.44**	0.21

*EF-OEU*	0.00	0.10	0.00	0.00	0.02	0.00	0.00	0.00	0.06	**0.46**	0.36

*EK-CA*	0.06	0.06	0.00	0.00	0.00	0.00	0.00	0.00	0.06	**0.67**	0.17

*EK-CC*	0.00	0.07	0.00	0.00	0.00	0.00	0.00	0.00	0.04	**0.50**	0.39

*EK-CI*	0.03	0.05	0.05	0.00	0.00	0.00	0.00	0.00	0.00	**0.46**	0.41

*EK-NA*	0.02	0.08	0.04	0.00	0.00	0.00	0.00	0.00	0.02	0.41	**0.43**

*IB-ANMWN*	0.00	0.06	0.03	0.00	0.00	0.00	0.00	0.00	0.03	0.36	**0.53**

*IB-EAEEUAE*	0.00	0.13	0.04	0.00	0.00	0.00	0.00	0.00	0.10	0.35	**0.38**

*IB-EUE*	0.00	0.08	0.04	0.00	0.00	0.02	0.00	0.00	0.06	**0.48**	0.32

*IB-IAAUA*	0.00	0.18	0.04	0.00	0.00	0.00	0.00	0.00	0.00	**0.50**	0.29

*IB-IEINOI*	0.00	0.12	0.00	0.00	0.00	0.00	0.00	0.00	0.00	**0.51**	0.37

*IB-IMIEI*	0.00	0.04	0.00	0.00	0.02	0.00	0.00	0.00	0.08	**0.52**	0.34

*IB-IOINO*	0.00	0.04	0.06	0.00	0.00	0.00	0.00	0.00	0.04	0.40	**0.46**

*IB-MNENN*	0.00	0.02	0.02	0.00	0.00	0.02	0.00	0.00	0.04	**0.56**	0.33

*IB-NEI*	0.00	0.13	0.06	0.00	0.00	0.00	0.00	0.00	0.08	**0.48**	0.25

*IB-OII*	0.02	0.08	0.04	0.02	0.00	0.02	0.00	0.02	0.00	**0.48**	0.32

*IB-ONMNI*	0.00	0.08	0.02	0.00	0.00	0.00	0.00	0.00	0.04	0.38	**0.48**

*IG-C*	0.00	0.07	0.01	0.00	0.01	0.01	0.00	0.00	0.06	**0.47**	0.36

*OR-AO*	0.00	0.11	0.00	0.00	0.00	0.00	0.00	0.00	0.00	**0.52**	0.37

*OR-ENEEAU*	0.00	0.04	0.05	0.00	0.01	0.01	0.00	0.00	0.04	0.40	**0.44**

*Cross River Grand Total*	0.00	0.08	0.03	0.00	0.00	0.00	0.00	0.00	0.04	**0.45**	0.38

											

*IG-E*	0.00	0.06	0.02	0.00	0.00	0.02	0.00	0.00	0.02	**0.69**	0.20

*IG-N*	0.00	0.04	0.00	0.00	0.00	0.00	0.00	0.00	0.29	**0.47**	0.20

*CA-BT*	0.03	0.06	0.06	0.00	0.00	0.00	0.00	0.00	**0.36**	0.18	0.30

*CA-FB*	0.00	0.09	0.04	0.00	0.01	0.00	0.01	0.00	0.07	**0.67**	0.12

*CA-WA*	0.00	0.04	0.00	0.00	0.00	0.00	0.00	0.00	0.03	**0.62**	0.31

*GH-AEW*	0.00	0.00	0.00	0.00	0.00	0.00	0.00	0.00	0.14	**0.52**	0.33

*GH-AKE*	0.00	0.00	0.08	0.00	0.00	0.00	0.00	0.00	0.31	0.24	**0.37**

*GH-ASWW*	0.00	0.00	0.00	0.00	0.23	0.00	0.00	0.00	0.18	0.27	**0.32**

*GH-EHVR*	0.02	0.02	0.05	0.00	0.00	0.00	0.00	0.00	0.11	**0.42**	0.38

*GH-FEWR*	0.03	0.00	0.02	0.00	0.02	0.00	0.00	0.00	0.30	0.25	**0.38**

*All Populations Total*	0.01	0.07	0.03	0.00	0.01	0.00	0.00	0.00	0.08	**0.46**	0.34

Modal NRY types shown in bold type.

Secondly the presence of E1b1a*, E1b1a7 and E1b1a8 haplogroups dominated the network but with substantial haplotype sharing among all three clades, consistent with a relatively recent common genealogical origin at the E1b1a root. One haplotype (15-12-21-10-11-13), which has previously been identified as a possible signature type for the expansion of the Bantu-speaking peoples [21-23] (though it is actually present at appreciable frequencies in other Niger-Congo speaking peoples as far west as Guinea-Bissau [22]), stands out as the most frequent and is predominantly found within E1b1a8. Examining each haplogroup separately [Additional file 1: Supplemental Figure S3] shows that E1b1a8 haplotypes are tightly clustered around this haplotype in a star-like manner while E1b1a7 is more diffusely spread with multiple high frequency haplotypes implying a longer evolutionary period since this haplogroup arose. This is reflected in the substantially lower Average Squared Distance (ASD) values for E1b1a8 compared to E1b1a7 [Additional file 2: Supplemental Table S7] (though, depending on the growth model used, the confidence intervals for the two haplogroups did overlap), which can be interpreted as younger Time to the Most Recent Common Ancestor (TMRCA) estimates [24] [Additional file 2: Supplemental Table S8]. E1b1a* (which was found at a slightly higher frequency in Ghana) is very diffuse with regard to microsatellite haplotypes, which suggests that further UEP delineation may be informative.

**Figure 4:**
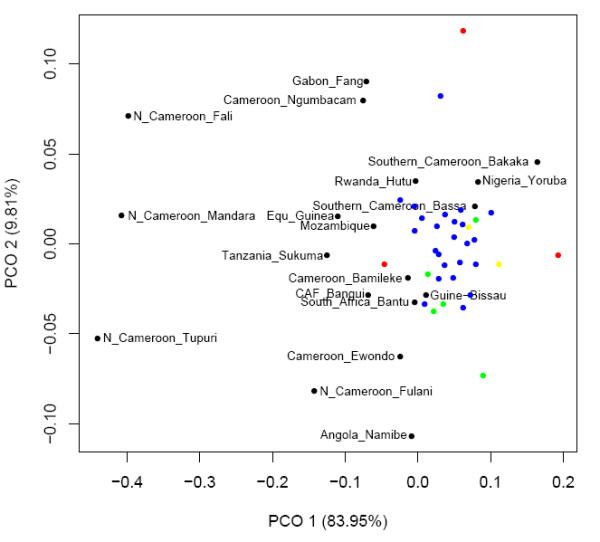
**PCO plot based on NRY 5 microsatellite R_ST _values for populations from the Cross River region (blue), Cameroonian NWP (red), Ghana (green), Igboland (yellow) as well sub-Saharan African populations collected in previous studies**.

We compared our West Central African data for 5 of 6 microsatellites to data from previous studies in sub-Saharan Africa (see Methods), included ethnic groups that were both geographically very close and distant to our own populations [Additional file 2: Supplemental Table S9]. Of the 19 ethnic groups compared (which included 9 Cameroonian and 1 Nigerian group), only 7 possessed a 5-microsatellite version of the potential Bantu signature haplotype. A PCO plot (Figure 4) based on R_ST _[Additional file 2: Supplemental Table S10a] showed ethnic groups from northern Cameroon and Gabon to be noticeably differentiated from all other sub-Saharan African population, a consequence of a high frequency of typically Asian NRY lineages [25]. With regard to the remaining populations, there was no clear correlation with geography though our West Central African population did demonstrate similarity with the majority of their geographic neighbours, while being slightly more differentiated from the geographically distant Angolan and Tanzania ethnic groups. However there was a somewhat unexpected difference with the Cameroonian Ewondo and Ngumbacam samples.

### mtDNA distribution

**Figure 5:**
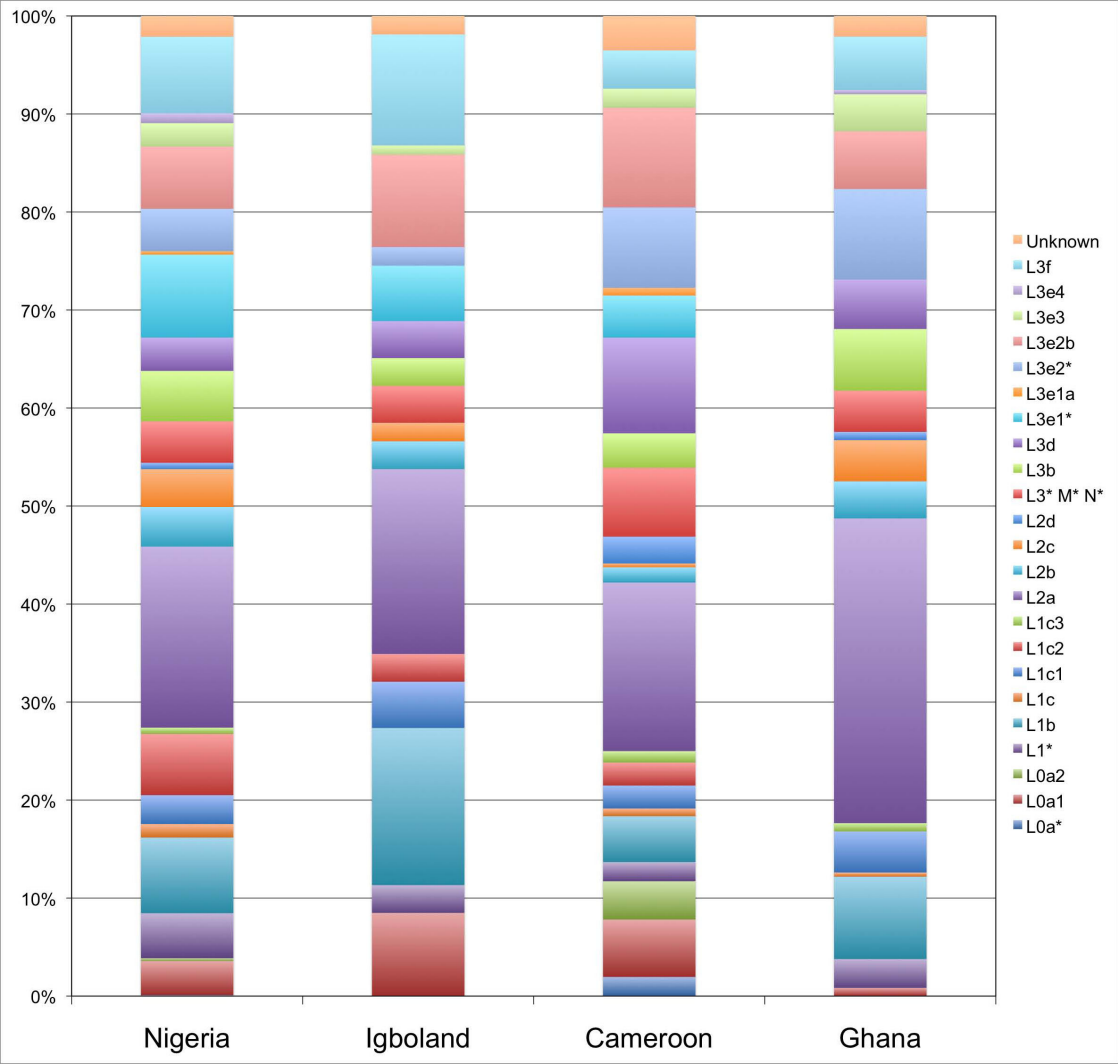
**mtDNA haplogroup frequencies in the Cross River regions, Cameroonian NWP and Ghana**.

Torroni et al. [26] have previously warned against the dangers of mtDNA haplogroup classification based solely on HVS-1 data. A median-joining network of all samples colour coded by their expected haplogroups as defined by Salas et al [27] [Additional file 1: Supplemental Figure S4] does demonstrate some assignment errors but in general good clustering around predicted haplogroups is observed. In addition the WTTI ratios (the ratio of the number of weighty transitions to the number of transversions plus indels) for the four populations considered (Cross River = 1.4, Igbo = 1.5, Cameroon = 1.2, Ghana = 3.1) were close to those previously reported for African datasets (Bandlet et al [28] = 1.5), which suggests the data presented here are reasonably problem-free. Typical of sub-Saharan Africa, L2a [27] is the most frequently observed haplogroup, though at substantially higher frequency in Ghana (see Figure 5). L3e is the most frequent L3 clade with L3e2 being predominant while other haplogroups that have previously been found in West Central Africa, such as L0a, L1b, and L1c, are all found at appreciable frequencies in our dataset. Interestingly, while present in the Cross River region and Cameroonian NWP, L3e1 is absent from Ghana, while L0a is found at a very low frequency.

**Figure 6:**
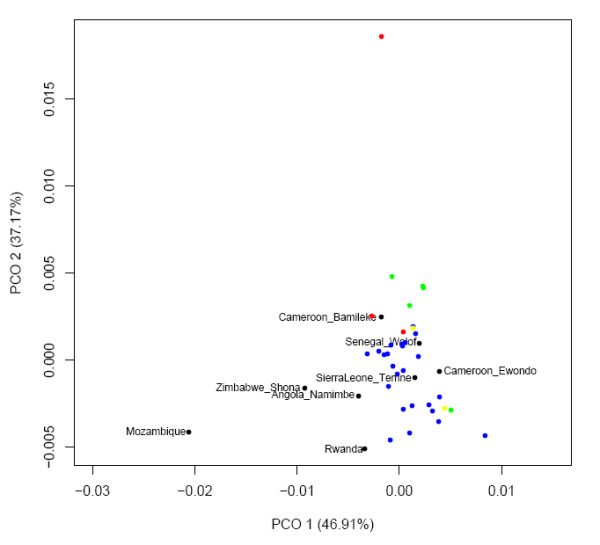
**PCO plot based on mtDNA HVS-1 F_ST _values for populations from the Cross River region (blue), Cameroonian NWP (red), Ghana (green), Igboland (yellow) as well sub-Saharan African populations collected in previous studies**.

Direct comparison to existing HVS-1 haplotype data [Additional file 2: Supplemental Table S11] via F_ST _values (Figure 6) [Additional file 2: Supplemental Table S10b], ignoring CA-BT (which had already been identified as an outlier [see Figure 3]), revealed a stronger geographic correlation in comparison to the NRY data, with a decent clustering of our West Central African populations to other Cameroonian groups and clear differentiation with samples from Angola, Rwanda, Zimbabwe and Mozambique. Interestingly, the more West African populations of Senegal and Sierra Leone grouped tightly with our populations.

## Discussion

### Cross River region homogeneity

The overall genetic homogeneity observed in the Cross River region was consistent with estimates of current gene flow derived from recent sociological data and demonstrates that major language differences, such as between Igbo and the Lower Cross languages, can be maintained in the presence of substantial gene flow over a significant period of time. However, the case presented here involves the majority languages spoken in the region. It remains to be seen whether such high levels of gene flow also apply to groups speaking less common languages (such as the Nkari of which there are less than 10,000 speakers [29]), where increased genetic isolation may aid (directly or indirectly) in conserving identity of the group. It is also notable that despite the populations in the region being primarily patrilineal, a lack of genetic structure was observed for both the NRY and mtDNA, though it is not possible to conclude whether this is due to equal male and female migration rates as the mutational properties of the NRY and mtDNA polymorphisms analysed are not directly comparable.

When the two Igboland groups were compared to the Cross River region clans a large proportion of pairwise comparisons between the two regions demonstrated significant differences. The Igboland groups, despite being in close proximity to each other, even demonstrated differences between themselves, suggesting perhaps that the Cross River region may be more homogenous than is typical for the broader region (and further fine-scale studies in other regions such as Igboland should be encouraged). One factor that may have contributed to the Cross River region's homogeneity was its position as a major slave post (additionally the region was already an important highway for inter-group commerce), which may have led to an unusually high level of inter-ethnic group mixing over as long as 200 years and thus significantly increased gene flow among speakers of different languages. Intriguingly some NRY haplogroups that are possibly (though further resolution would be required) indicative of European ancestry (P*(xR1a), J and possibly F, G, and I) are found at very low frequencies amongst the Cross River samples and may have entered the Cross River gene pool as a consequence of male introgression of slave traders.

Some caution must be exercised in over-interpreting the data presented here. Mantel and partial Mantel tests did reveal, albeit with a moderate P-value, a significant correlation between genetic and linguistic distance at the NRY UEP+MS FST level in the Cross River region. It could be suggested that, at least for the NRY, further microsatellite typing may eventually differentiate the apparently homogenous Cross River region and our results simply reflect a lack of marker resolution. However, given the large number of UEP+MS and HVS-1 haplotypes in our dataset, including a number of singletons, it seems unlikely that the allele frequencies amongst the different populations would not have drifted apart over a number of generation without gene flow being a major force within the Cross River system, as demonstrated by the simulations conducted to examine the effect of gene flow on population genetic structure. Increasing the marker resolution would certainly help differentiate individuals (important for tracking migration routes) but not necessarily populations and are unlikely to aid in measuring gene flow within a particular system of populations. The clear interpretability of our results also help justify the continued use of uniparental genetic systems when investigating demographic history, the advantages of which have previously been described by Underhill and Kivisild et al. [30].

### West Central African differentiation

When the Cross River region was analysed alongside the Cameroonian NWP and Ghana strong genetic differentiation was observed between all three regions at all NRY and mtDNA levels. The level of differentiation is somewhat reduced for evolutionary deeper analysis such as at the NRY UEP level, as observed by the high E1b1a*, E1b1a7 and E1b1a8 frequencies in all three regions, while the increased differentiation observed at finer scales of genetic resolution is a result of, as expected, highly restricted (if not non-existent) gene flow more recently due to the large geographic distances involved. However it is also appears that a simple isolation by distance model is not adequate to fit the pattern observed.

Despite being geographically much closer, the Cameroonian NWP populations are noticeably more differentiated from the Cross River region than Ghanaian populations, as seen clearly seen in the PCO plots (Figure 3), with the Cameroonian NWP populations demonstrating the greatest differentiation both between each other and non-Cameroonian populations at the NRY UEP+MS F_ST _and mtDNA F_ST _levels. Linguistically the distance between the Cross River region and both the Cameroonian NWP and Ghana (at least for the particular languages considered in this study) is much less pronounced than the corresponding geographic distances. As a consequence Mantel and partial Mantel tests show a stronger correlation between genetic and linguistic distance at finer genetic resolutions. However, the meaningfulness of these correlations is somewhat questionable, given that while the broad relationships of the languages considered here are generally accepted, the lexicostatistics that the actual distances between languages are based on are at best a first estimate with numerous potentially problematic approximations [Additional file 1: Supplemental Section 3]. While both are clearly involved (and likely confounding) at some level, not until more reliable language distance estimates are generated can the relative contributions of geography and language to genetic divergence amongst these West Central African populations be assessed.

The substantial amount of genetic differentiation within the Cameroonian NWP may be driven by the extreme topography of the region, which is a largely highland area with many valleys, hills and mountains (Mount Oku is located in the NWP and is the second highest mountain in West Central Africa) and thus presents significant physical barriers to gene flow between neighbouring populations. As the rate of linguistic separation may well also be increased by such physical barriers it is possible that at smaller geographical scales where the topography is particularly varied, language will be a better guide to genetic differentiation than geography alone, though the desire to maintain a separate identity within close quarters is also likely to major force for shaping genetic heterogeneity.

### E1b1a8 and the expansion of Bantu-speaking peoples

Though not the primary focus of the study, the typing of the U175 marker [31] permits important new insights into the demographic processes influencing haplogroup E1b1a. While none of the populations studied here are Narrow Bantu speakers, the star-like network of E1b1a8, especially in comparison to E1b1a7, coupled with a recent TMRCA based on the level of haplogroup specific microsatellite diversity of 1866-2355 years [Additional file 2: Supplemental Table S8] (though the authors recognise that TMRCAs do not necessarily correlate with demographic events) hint at men with NRY that belong to this subclade playing a prominent role in the expansion of the Bantu-speaking peoples. This possibility is further reinforced by the haplotype that has been observed at high frequencies amongst Bantu-speaking populations, including South Africa (the putative Bantu signature haplotype [21]) being observed almost exclusively within E1b1a8 in our dataset. Thus further typing of U175 in other Bantu-speaking populations along both streams of the proposed expansion may yield important clues to the movement of Bantu-speaking farmers.

Our Cross River and Cameroonian NWP datasets are located adjacent to the proposed source of proto-Bantu and their similarity for the NRY to other populations both neighbouring and more distant demonstrates the potential impact of the expansion of Bantu famers in homogenising the NRY profile of sub-Saharan Africa. For example, the South African Bantu speakers are barely more differentiated from our West Central African dataset than the Bamileke. This pattern is in contrast to that seen for mtDNA, where our West Central African populations are more easily differentiated from the more geographically distant southern African populations, consistent with previous data [19] that suggests a more gradual and short range movement of female lineages than men during this migration period. Haplogroups L0a, L1c, L2a, L3e and L1e have all been associated with the expansion of Bantu-speaking farmers [19] (the origin of L2a has actually been proposed to be from the Cameroonian Plateau) and their substantial presence in our Cross River and Cameroonian NWP datasets, and in some circumstances absence from the more westerly Ghanaian dataset (such as L3e1, which is very common in southeastern Africa), certainly add weight to these claims.

## Conclusion

In this study we have been able to elucidate that languages and peoples can move independent of each other within the Cross River region of Nigeria, a finding that will be of considerable interest to linguists working on aspects of language contact. A major reason we have been able to gain insight at such a fine geographic scale is the quality of the dataset assembled. There has, unfortunately, been a tendency when examining African genetic diversity to utilise datasets of small size with samples of undeclared origin and relationships. The practice of assembling dense DNA sample sets of known and detailed provenance, as previously called for by anthropologists and linguists [32], will be the most vital aspect when conducting studies to answer the many complex questions likely to be encountered in the course of unravelling demographic histories of geographically restricted African ethnicities.

## Methods

### Sample collection procedure

Buccal swabs were collected from males over eighteen years old unrelated at the paternal grandfather level from locations in South East Nigeria as shown in Table 1. All buccal swabs were collected anonymously with informed consent. Ethical approval was obtained from University College Hospitals and University College London Joint Committee on the Ethics of Human Research (reference number 99/0196). Sociological data were also collected from each individual including age, current residence, birthplace, self-declared cultural identity, first language, second language and (when available) clan affiliation (Clan identities were verified with information presented in Cross River and Akwa Ibom State Population Bulletin 1982-90 [33]) for the individual as well as similar information on the individual's father, mother, paternal grandfather and maternal grandmother. The samples were classified into groups primarily by first language spoken, then by place of collection and thirdly, when available, by clan or some other subsidiary criterion. Where collections from a particular group were made in more than one location (for example the Ediene Abak were collected from two neighbouring villages: Afaha Esang and Ikot Ubom) and co-ordinate data are available for both sites, locations are represented by averages.

Buccal swabs and similar sociological data as described above were also collected from males eighteen years or older unrelated at the paternal grandfather level from the following groups:

CA-BT: Tikar speakers from Bankim Cameroon (n = 34), CA-FB: Bamoun speakers from Foumban Cameroon (n = 117), CA-WA: Aghem speakers from Wum Cameroon (n = 118), GH-AEW: Twi speakers from Enchi Ghana (n = 21), GH-AKE: Twi speakers from Kibi Ghana (n = 51), GH-ASWW: Twi speakers from Sefwi Wiawso Ghana (n = 22), GH-EHVR: Ewe speakers from Ho Ghana (n = 88), GH-FEWR: Fante speakers from Enchi (n = 61).

Standard phenol-chloroform DNA extractions were performed on all samples.

### Assembly of comparison NRY and mtDNA datasets

NRY data for 5 microsatellites (DYS19, DYS390, DYS391, DYS392, DYS393) was assembled from previous studies conducted on sub-Saharan African populations for comparison to data generated in this study. The populations considered were Namibe from Angola [34]; Bangui from the Central African Republic [35]; Ngumbacam [36], Bamileke[37] and Ewondo [37] from Cameroon; Fali [38], Fulani [38], Mandara [38] and Tupuri [38] from Northern Cameroon; Bakaka [38] and Bassa [38] from Southern Cameroon; individuals from Equatorial Guinea [39]; Fang from Gabon [36]; individuals from Guinea' Bissau [40]; individuals from Mozambique [22]; Yoruba from Nigeria [41]; Hutu from Rwanda [37]; Bantu speaker from South Africa [21]; and Sukuma from Tanzania [41].

HVS-1 VSO haplotype data from positions 16030 to 16360 was also assembled from previous studies from the following populations: Namimbe from Angola [34]; Bamileke [42] and Ewondo [42] from Cameroon, individuals from Mozambique [43]; Hutu from Rwanda [44]; Wolof from Senegal [45]; Temne from Sierra Leone [46]; and Shona from Zimbabwe [44].

### Y-chromosome typing

The NRY of all South East Nigerian samples as well as all Cameroonian and Ghanaian samples were typed in the following manner: standard TCGA kits were used to characterise six microsatellites (DYS19, DYS388, DYS390, DYS391, DYS392, DYS393) and eleven biallelic Unique Event Polymorphism (UEP) markers (92R7, M9, M13, M17, M20, SRY+465, SRY4064, SRY10831, sY81, Tat, YAP), as described by Thomas et al. [47]. Microsatellite repeat sizes were assigned according to the nomenclature of Kayser et al. [48]. Where necessary the additional markers M191 and U175, were typed using a tetra primer ARMS PCR method [49]. Each PCR involved four oligonucleotide primers and resulted in the amplification of a full fragment (control band) and one allele specific fragment (see supplementary materials for further details [Additional file 2: Supplemental Table S12]). P12f2 was typed as described by Rosser et al. [11]. NRY Haplogroups were defined by the 14 UEP markers according to the nomenclature proposed by Karafet et al. [50] [Additional file 1: Supplemental Figure S5]. Markers typed were chosen to reflect that as well as characterising NRY types of recent African origin we would also be likely to characterise a minority of NRY types of recent European origin due to possible introgression from North Atlantic slave traders.

### mtDNA typing

The mtDNA (Hypervariable Segment 1) HVS-1 region of all South East Nigerian samples as well as all Cameroonian and Ghanaian samples was sequenced as described by Veeramah et al. [51]. HVS-1 Variable Site Only (VSO) haplotypes were determined for all samples from South East Nigeria by comparing sequence data covering nucleotides 16020-16400 with the Cambridge Reference Sequence [52,53]. Haplotypes were defined by base changes and nucleotide positions where substitutions, insertions or deletions occurred. Tentative mtDNA Africa-specific haplogroup classification was based on the scheme of Salas et al. [27]. HVS-1 VSO haplotypes were also determined for all samples from Cameroon and Ghana with sequence data covering nucleotides 16023-16380. South East Nigerian HVS-1 coverage was reduced to this range for comparisons including these groups.

### Statistical and population genetic analysis

Genetic differences between pairs of populations when individuals in populations were characterised by a) NRY UEP haplogroups, b) combined NRY UEP haplogroup and six microsatellite haplotypes (UEP+MS) or c) mtDNA HVS-1 VSO haplotypes were assessed using an Exact Test of Pairwise Population Differentiation (ETPD) with 10,000 Markov steps [54,55].

Population Genetic Structure was estimated using Hierarchical Analysis of Molecular Variance (AMOVA) [56] based on a particular mutation model to generate a single Fixation Index statistic, F_ST_, when a simple structure of populations within a single group was defined, or three Fixation Indices, F_ST _(the within-population Fixation Index), F_SC _(the among-populations within-group Fixation Index) and F_CT _(the among-group Fixation Index), when a more complex structure of populations within multiple groups was defined. Significances of Fixation Indices are assessed by randomly permuting individuals (given that only haploid systems are considered) among populations or groups of populations, depending on the Fixation Index being tested and after every round of permutations, of which 10,000 were performed, Fixation Indices are recalculated to create a null distribution.

Population pairwise genetic distances were estimated from Analysis of Molecular Variance φST values [56]. The genetic distances used were a) F_ST _[57] (when individuals in populations were described by UEP haplogroups, UEP+MS haplotypes and mtDNA HVS-1 VSO haplotypes), b) R_ST _[58] (when NRY were characterised by the six microsatellites) and c) the Kimura-2 parameter model (which allows different transition and transversion rates) with gamma distribution of value 0.47 (K2) [59] (when mtDNA was characterised by HVS-1 sequences with gaps removed). Significance of genetic distances was assessed by permutation of individuals as described above for testing significance of Fixation Indices. All the above was performed using Arlequin software [60].

Principal Coordinates Analysis (PCO) [61] was performed using the 'R' statistical package http://www.R-project.org by implementing the 'cmdscale' function found in the 'mva' package on pairwise F_ST _(or equivalent) matrices.

TMRCA estimates based on the level haplogroup specific microsatellite diversity and associated confidence intervals (CIs) were estimated using YTIME software [62]http://www.ucl.ac.uk/tcga/software/index.html. An inter-generation time of 25 years was applied to convert from generations to years. A mutation rate of 0.002 [63] was utilized under a single-stepwise mutation model and under a length-dependent mutation model the constants *a *and *b *in the equation *μ *= *a *+ *bL *were represented by -0.004758677 and 4.46E-04 respectively (YTIME user guide http://www.ucl.ac.uk/tcga/software/index.html). The most frequent haplotype in the corresponding haplogroup was utilized as the ancestral haplotypes (therefore this method does not take into account error in the choice of ancestral haplotypes in the genealogy).

Mantel and Partial Mantel tests [64] were performed between genetic distance and both geographic and linguistic distance using the 'R' package 'Vegan', which uses the Pearson product-moment method. Significance was assessed by permuting the rows and columns of the matrices 1,000 times.

Geographic distances were Great Circle distances estimated from latitude and longitude data. Linguistic distances were constructed as described in the supplementary materials [Additional file 1: Supplemental Section 3], drawing from lexicostatistics reported in the literature and incomplete data matrix prediction algorithms.

Median Joining Networks were constructed for NRY data as described by Helgason et al. [65] and for mtDNA data as described by Vilar et al. [66].

NRY and mtDNA simulations were performed as described in the supplementary materials [Additional file 1: Supplemental Section 2], the results of which could be compared to empirical data in order to guide our understanding of the effect migration rate and sample size on genetic structure in the Cross River region. These simulations are at best crude approximations of the true Cross River region system that do not explore the full likely parameter space and thus are not formally statistically assessed in comparison to our observed data.

## Authors' contributions

KRV drafted the manuscript, participated in conceiving and the design of the study and performed the majority of analysis. BC provided the linguistic and historical background and participated in conceiving and the design of the study. NAP performed the M191 and U175 NRY typing. AP wrote the Python code for the NRY and mtDNA simulations. CP performed the Network analysis. DZ participated in conceiving and the design of the study. NM aided in the statistical analysis. MW participated in conceiving and the design of the study and aided in the statistical analysis. NB participated in conceiving and the design of the study and helped draft the manuscript. MG participated in conceiving and the design of the study and helped draft the manuscript. All authors read and approved the final manuscript.

## Supplementary Material

Additional file 1 **Supplemental Sections and Figures**. A document file containing Supplemental Sections 1-3 and Supplemental Figures S1-S12. Click here for file

Additional file 2 **Supplemental Tables**. A spreadsheet file containing Supplemental Tables S1-S14. Click here for file
