# Supplementary material for: Little genetic differentiation as assessed by uniparental markers in the presence of substantial language variation in peoples of the Cross River region of Nigeria
Source: BMC Evol Biol. 2010 Mar 31;10:92. doi: 10.1186/1471-2148-10-92 (PMC2867817; doi:10.1186/1471-2148-10-92)

Little genetic differentiation as assessed by uniparental markers in the presence of substantial language variation in peoples of The Cross River region of Nigeria: Supplemental Information

Supplemental Section 1: Cross River history expanded

The resident peoples of the region have been characterised as 'Syncretic Christian'; that is to say, nominally Christian but retaining aspects of traditional animist worship. In general their social structures are 'acephalous' (lacking a fixed, centralised political structure). Although the Efik ethnic group do have a 'king', or paramount ruler - the Obong - the power and role of the Obong is not equivalent to that of, say, the fon in Grassfields societies of Cameroon. The social characteristics of the major groups in the region tend to be of an exogamous, patrilocal and patrilineal nature[1].

While movements of peoples in various directions are indicated, they, in general, relate an expansion southward, perhaps in search of trade opportunities with the newly arrived Europeans (Latham [1], citing reports of Europeans from this period, concludes that the place that has since become Calabar was only settled after the first contact with Europeans) but more likely due to pressure from expanding Igbo populations.

[1] Information on Lower Cross groups: Anaang, Efik, Ibibio, and Oro is from Forde and Jones (1950), Udo (1983) and Uya (Uya 1984); Efut: from Connell (1983); Ejagham: Talbot (1912); Igbo: Basden (1966) and Forde and Jones (1950). In all cases information has been supplemented from Connell unpublished field notes (1983).

A village or region named ‘Ibom’ is often suggested as a point of origin. There is today a village called Ibom near the Igbo town of Arochukwu that is situated in the northwest of the Cross River area, in the border region between Igbo and Ibibio territories. An alternative account suggests dispersal from the Ibom Arochukwu area was a response to conflict with the expanding Igbo speaking population. However it should be noted that most Lower Cross traditions deal with the relatively recent past in comparison to these oral traditions discussed.

Several of the Lower Cross groups also have diverging traditions, for example of having migrated from Cameroon. The Efik, in particular, have a variety of conflicting traditions, which are summarised in Noah [2]. Among them is a claim of origin in, and migration from, ancient Palestine [3]. This story tells of a migration from the Middle East via Sudan, Chad and Benin, with stops among the Igbo and then Ibibio, and the founding of Calabar. However some versions of the account claim no more than that the Efik are of Igbo origin. Most of the Efik traditions have as a common thread a final stop among the Ibibio, specifically in the Uruan area. The Hart Commission [4] investigated various Efik claims and essentially concluded that they were without foundation. The report concluded:

> “The last tribes among whom the Efiks might have lived were the Ibibio. If they had lived among the Ibo [sic.] and were in fact Ibo [sic.] in origin, there is no means ready to hand to determine the truth or falsity of this claim of origin.”

The Efut, another group found within the boundaries of Calabar, claim an origin in a Bantu-speaking area to the east of the Cross River estuary in Cameroon. It is claimed by some that their language was once Londo, a Northwest Bantu language (A11-according to the nomenclature of Guthrie [5]) which is still spoken in Cameroon [6,7], but they have since adopted Efik as their primary tongue.

The oral traditions of the Ejagham, also known as Ekoi, are less well documented. The main body of the Ejagham population is to be found in the Upper Cross River basin and extends southward. One Ejagham subgroup (also known as ’Qua’ or ‘Ekin’) occupies a part of Calabar, and claim to have arrived there before the Efik, having migrated southward from the main Ejagham area [2]. This claim is supported by the practice, continued to the present day, in which the Efik pay tribute to the Qua [4].

The Igbo constitute the third largest ethnic group in Nigeria, numbering (approximately) 18,000,000 [8]. They occupy much of the southeast of the country, forming an arc around the Cross River region. The Igbo are well known as traders and merchants and are found in every major urban area in Nigeria, including a sizeable population in Calabar. Many Igbo were brought to Calabar during the era of the slave trade. In more recent times, many others have settled and established businesses.

The Igbo are a relatively diverse group and from the linguistic standpoint comprise over 20 different lects [9]. Their oral traditions broadly speak of a north to south expansion

[10]. This expansion may still be in progress since only in relatively recent times has a sizeable Igbo population settled in the coastal areas of the Niger Delta.

Supplemental Section 2: Population demographic simulations

Methods

A simulation of Y and mtDNA population dynamics was designed as follows:

In each simulation a number of populations are placed randomly in a simulated world and connected by stochastic Gaussian random-walk migration, in which male and female individuals are allowed to migrate at different parameterized rates. The size of each population is Normally distributed ~N(1000, 50), and the initial NRY and mtDNA allele frequencies in all populations are set to a randomly generated distribution. In every generation each female individual has offspring, the number being Poisson distributed with a parameterized mean, and with the male:female ratio binomially distributed with mean 0.5. These offspring replace the adults in every population and individuals are then allowed to migrate between populations with sex-specific rates. The simulation proceeds for 100 generations, taking 50 samples of NRY and mtDNA allele frequencies from the male population at the end of every generation. Given the total period being examined during any simulation is relatively short, and that haplotype diversity is already set relatively high, mutation is not considered during the population history. Each generation pairwise ETPD, pairwise AMOVA-based F_{ST}s and AMOVA based Fixation indices analysis were applied to all possible pairs of populations present in the world for both

NRY and mtDNA types. This analysis was performed using Genepop-v4 [11] via implementation as a pipeline in software written by Adam Powell and modified by Krishna Veeramah (available from Krishna Veeramah by request). Though parameters were varied for testing purposes the default settings used unless otherwise stated were; generations = 100, mean number of offspring per female = 2.0, number of male alleles = 100 (reflecting high NRY UEP+MS haplotypes diversity), number of female alleles =100 (reflecting high mtDNA HVS-1 haplotype diversity), number of populations = 10, population size mean = 1000, population size standard deviation = 50, male migration rate = 0.1, female migration rate = 0.1, sample size from each population statistical analysis = 50 (reflecting general sample sizes observed in the Cross River dataset).

Results

ETPD

Results examining the length of time in generations that significant population pairwise differences persist [Additional file 1: Supplemental Figure S6] demonstrated that after a sharp drop of 60-10 generations from a migration rate interval of 0.0-0.06, the time quickly steadies at low values with a range of 3-1.3 generations at migration rates between 0.1-0.3. However sample size has a strong effect on these results [Additional file 1: Supplemental Figure S7] with higher sample sizes resulting in longer persistence times, especially at lower migration rates, though it should be noted that at migration rates of around 0.2 and onwards the effect of differing sample sizes is barely noticeable.

Differential rates of migration between sexes [Additional file 2: Supplemental Table S13a] show that high migration rates in one sex barely if at all affect the ETPD results in the other sex i.e. high migration rates in males do not lead to increased homogeneity in females when they themselves have low migration rate.

AMOVA based Fixation Indices

Much like the ETPD results average AMOVA based Fixation Indices values (across individual generations) [Additional file 1: Supplemental Figure S8] quickly decrease from relatively high values (0.1) to low values (0.02) within a small window (migration rates 0-0.04) before once again levelling off somewhat at a steady range (0.02-0.004 generation at migration rates of 0.04-0.3)). Interestingly observed Fixation Indices values for UEP+MS NRY haplotypes (-0.001) and HVS-1 mtDNA haplotypes (0.000) are still lower than the simulated values even for a migration rate as high as 0.3 migrants per generation. Unlike ETPD results AMOVA Fixation Indices are completely unaffected by sample size effects [Additional file 1: Supplemental Figure S9] and once again, differential geneflow between sexes has no immediate effect [Additional file 2: Supplemental Table S13b].

Pairwise F_{ST} results

Unlike the results for persistence of significant pairwise differences and global AMOVA F_{ST} results, the decrease in the frequency of significant pairwise genetic distances with increased migration rate is much more gradual [Additional file 1: Supplemental Figure

S10]. However, even at a migration rate of 0.3 the frequency of significant genetic distances (0.2) is still substantially less than observed in our observed data for for UEP+MS NRY haplotypes (0.05) and HVS-1 mtDNA haplotypes (0.06). Sample size does have a substantial effect on this metric [Additional file 1: Supplemental Figure S11] though even at n=25 it still does not reach our observed values. Differential geneflow between sexes again has no noticeable impact [Additional file 1: Supplemental Table S13c].

Supplemental Section 3: Generation of linguistic pairwise distances

Lexicostatistic similarity percentages [Additional file 2: Supplemental Table S14a] were compiled using the following sources: the pairwise values for the Lower Cross languages (Anaang, Efik, Ibibio and Oron) were taken from Connell & Maison [12]. No lexicostatistic similarity percentages were available for Ejagham languages in comparison with the other five Cross River region languages. Therefore data for three other Bantoid languages Tunen, Mambila (which represents different branches of Bantoid spoken near to the Cameroon-Nigeria borderland) and Bobangi (a Southern Bantu language spoken in the Democratic Republic of Congo) were used as surrogates for Ejagham as lexicostatistic similarity percentages had been calculated for these languages in comparisons between Efik and Igbo as well as each other by Schadeberg [13]. The pairwise value between Akan and Ewe is from Schadeberg [13], Asante being a particular dialect, representing Akan. The pairwise value between Ekoid (The Ekoid language in question being Nkim, not Ejagham) and Mambila is from Piron [14]. The pairwise value

between Aghem and Tikar is from Piron [15]. The pairwise comparisons between Tikar and Mambila and Tikar and Tunen are from Piron [16]. No suitable lexicostatistics were available for Bamoun so its similarity to Aghem (both are Narrow Grassfields groups) was estimated on the assumption that the similarity is larger than the average similarity between the three Southern Bantoid languages Tunen, Tikar, Bobangi but smaller than the average similarity between Oron and the three Lower Cross languages.

An incomplete lexicostatistic distance matrix was then calculated for the six Cross River, three Cameroonian and two Ghanaian languages used in this study by subtracting the lexicostatistic similarity percentages from 100% as performed by Weng and Sokal [17], with cells containing Ejagham pairwise comparisons found by taking the average lexicostatistic dissimilarity for the appropriate Tunen, Mambila, Bobangi and Nkim pairwise comparisons. Missing data (indicated by a question mark) in the distance matrix [Additional file 2: Supplemental Table S14b] were then estimated using the weighted least-square approach of Makarenkov and Lapointe [18] via the T-Rex software package (http://www.labunix.uqam.ca/~makarenv/trex.html) to give the final linguistic distance matrix [Additional file 2: Supplemental Table S14b]. The neighbor joining tree generated by this distance matrix [Additional file 1: Supplemental Figure S12] is of similar structure to that proposed by other sources such as the Ethnologue [8].

References

1. Latham AJH: **Old Calabar**. In *The impact of the international economy upon a traditional society* Oxford: Clarendon Press; 1973:1600-1891.

2. Noah ME: *Old Calabar: The City States and the Europeans*. Calabar: Scholars' Press; 1980

3. Akak EO: *The Palestine Origin of the Efiks*. Calabar: Akak and Sons; 1986

4. Hart AK: *Report of the Enquiry into the Dispute over the Obongship of Calabar*. Enugu: Government Printer; 1964

5. Guthrie M: *Comparative Bantu: an introduction to the comparative linguistics and prehistory of the Bantu languages*. Farnborough: Gregg Press; 1967

6. Connell B: **Unpublished fieldnotes**. 1983,

7. Thompson RF: *Flash of the Spirit: African & Afro-American art & philosophy*. New York: Vintage; 1983

8. Lewis MP: *Ethnologue: Languages of the World*. Dallas, Texas: SIL International; 2009

9. Manfredi V: **Igboid**. University Press of America; 1989:337-358.

10. Forde D, Jones GI: *The Ibo and Ibibio-speaking Peoples of South-eastern Nigeria*. London: Oxford University Press; 1950

11. Raymond M, Rousset F: **GENEPOP (version 1.2): population genetics software for exact tests and ecumenicism**. *J Heredity* 1995,248-249.

12. Connell B, Maison KB: **A Cameroun homeland for the Lower Cross languages?** *Sprache und Geschichte in Afrika* 1994,47-90.

13. Schadeberg TC: **The lexicostatistic base of Bennett & Sterk's reclassification of Niger-Congo with particular reference to the cohesion of Bantu**. *Studies in African Linguistics* 1986,69-83.

14. Piron P: **Identification lexicostatistique des groupes bantoïdes stables**. *Journal of West African Languages* 1995, **2**:3-39.

15. Piron P: **Classfication interne du groupe bantöide**. 1995,

16. Piron P: **Internal classification of the Bantoid language group, with special focus on the relation between Narrow Bantu, Southern Bantoid and Northern Bantoid**. In *Language History and Linguistic Description in Africa* Edited by Maddieson I, Hinnebusch T.J. Trenton N.J.: Africa World Press; 1998:65-74.

17. Weng Z, Sokal RR: **Origins of Indo-Europeans and the spread of agriculture in Europe: comparison of lexicostatistical and genetic evidence**. *Hum Biol* 1995, **4**:577-594.

18. Makarenkov V, Lapointe FJ: **A weighted least-squares approach for inferring phylogenies from incomplete distance matrices**. *Bioinformatics* 2004, **13**:2113-2121.

Supplemental Figure S1: Percentage of pairwise significant differences (P<0.05) of Cameroonian NWP and Ghanaian populations with Cross River clans at various NRY and mtDNA levels.

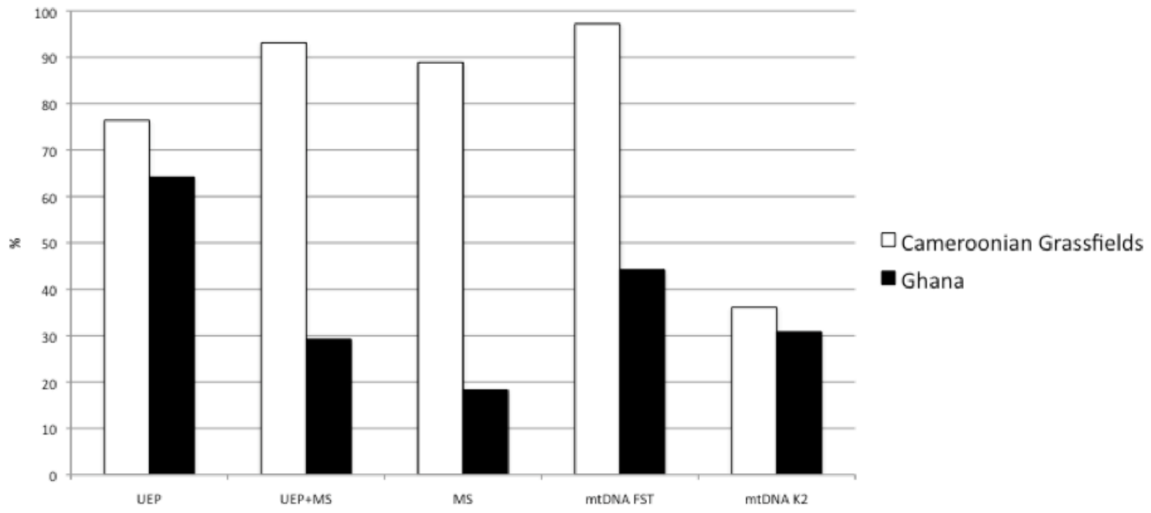

Supplemental Figure S2: Median joining network based on NRY microsatellite haplotypes for all Cross River region samples excluding singletons.

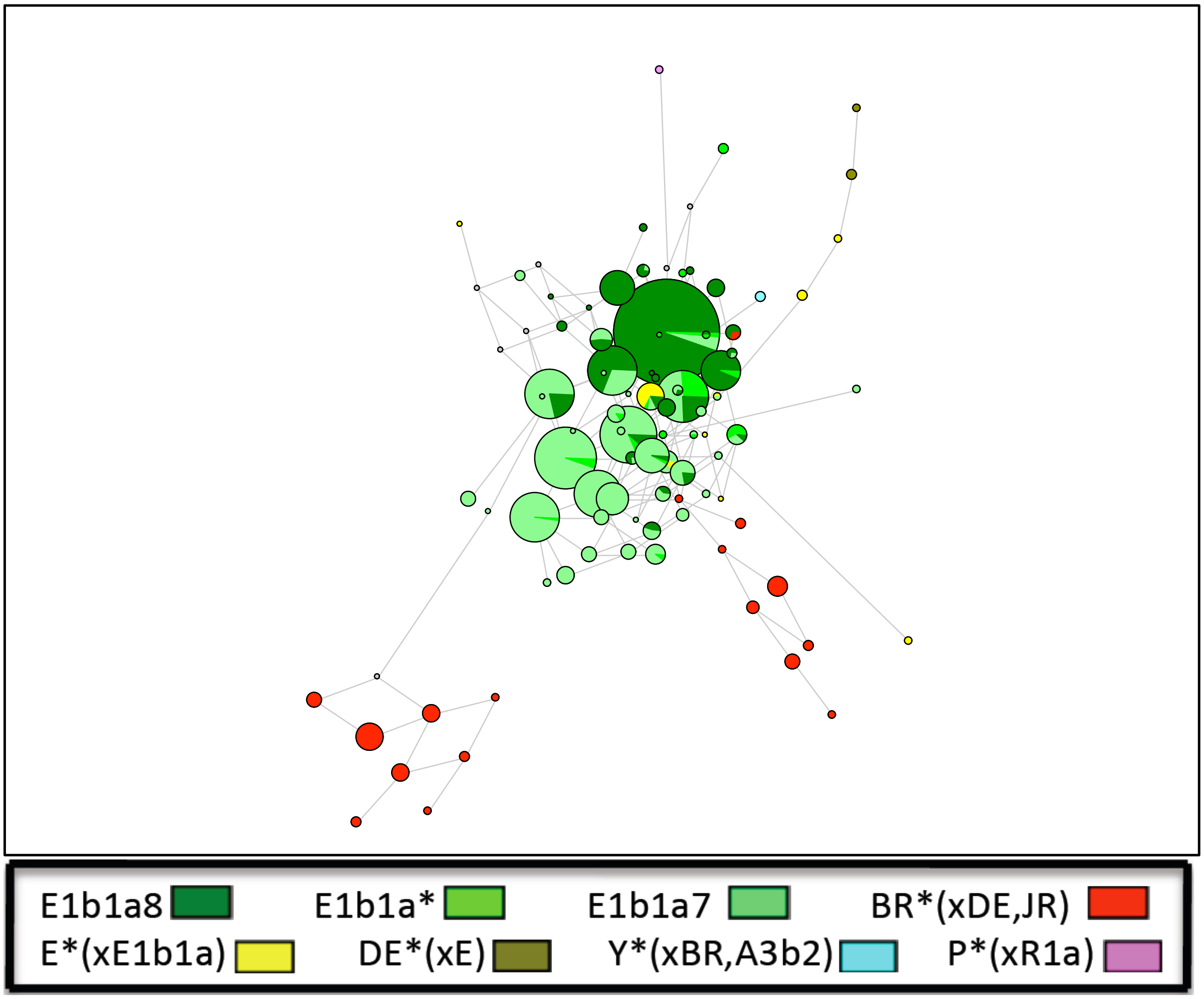

Supplemental Figure S3: Median joining network based on NRY microsatellite haplotypes for all Cross River region, Igboland, Cameroonian NWP and Ghanaian samples excluding singletons within a) E1b1a*, b) E1b1a7 and c) E1b1a8.

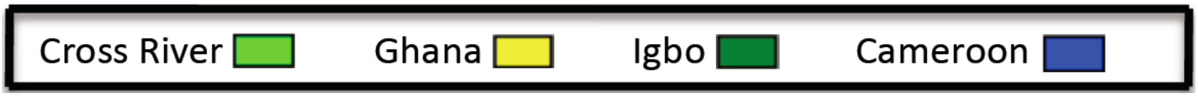

a)

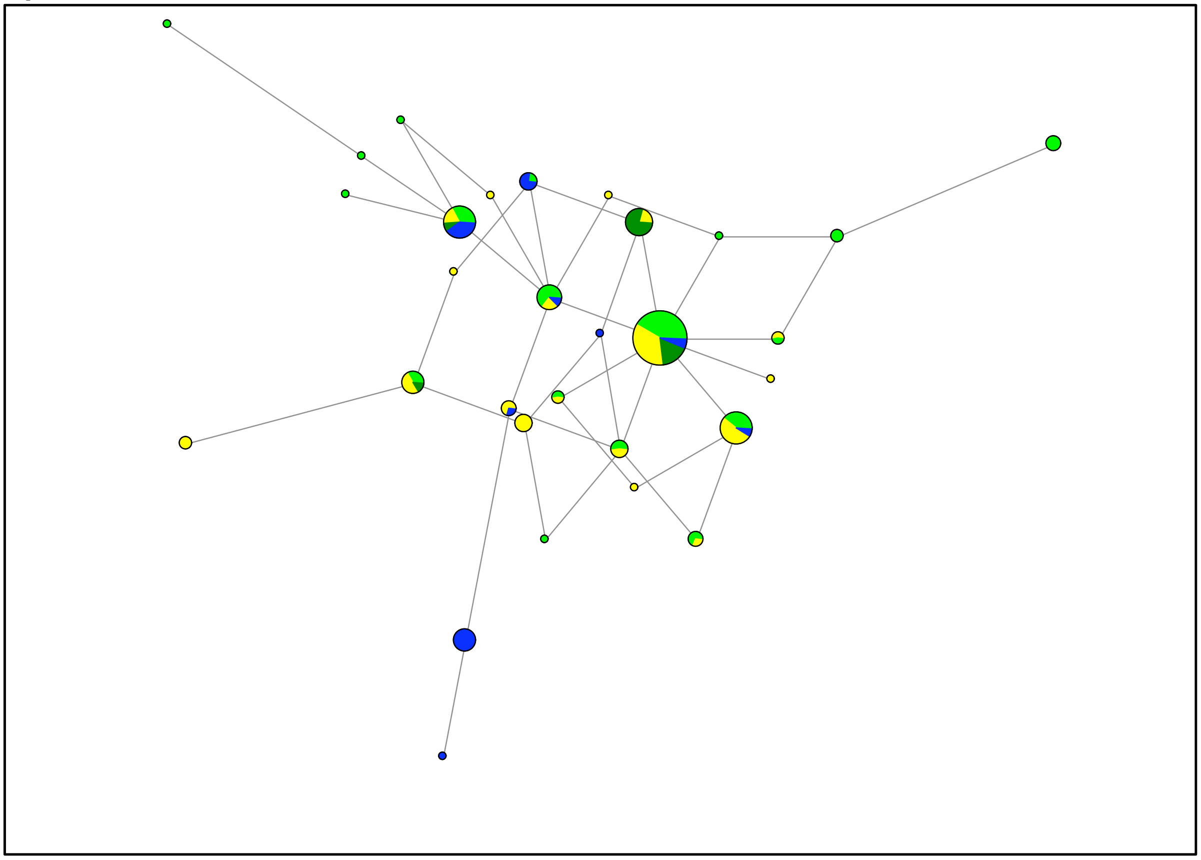

b)

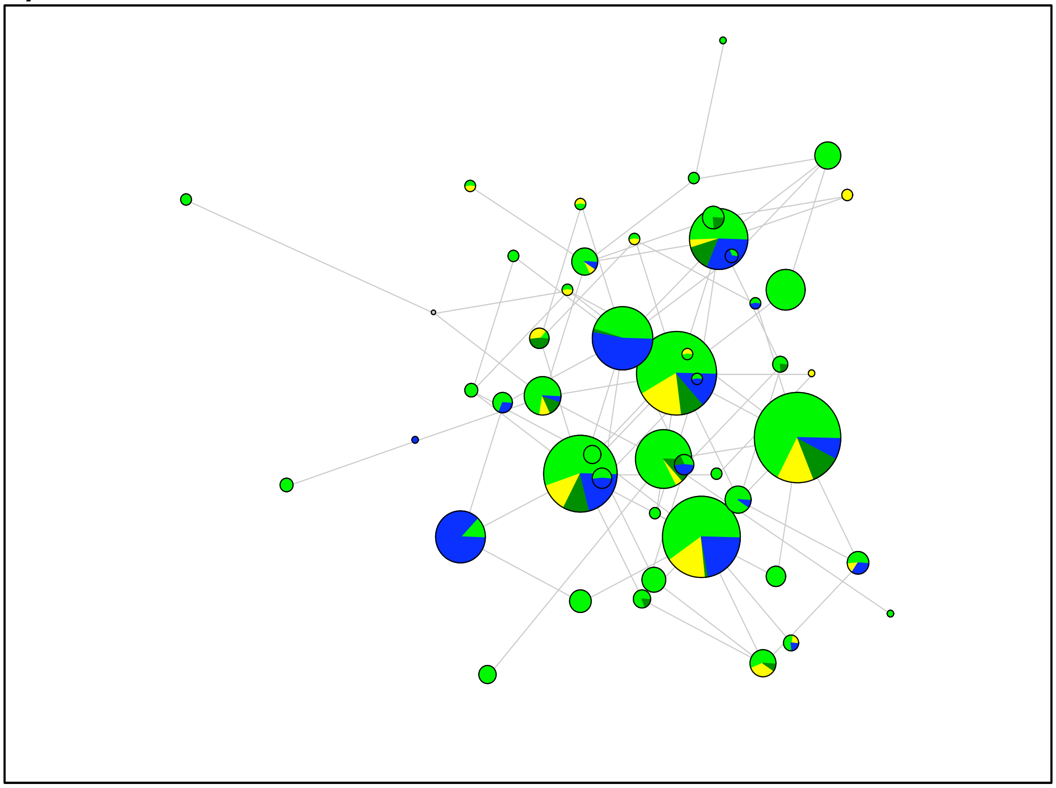

c)

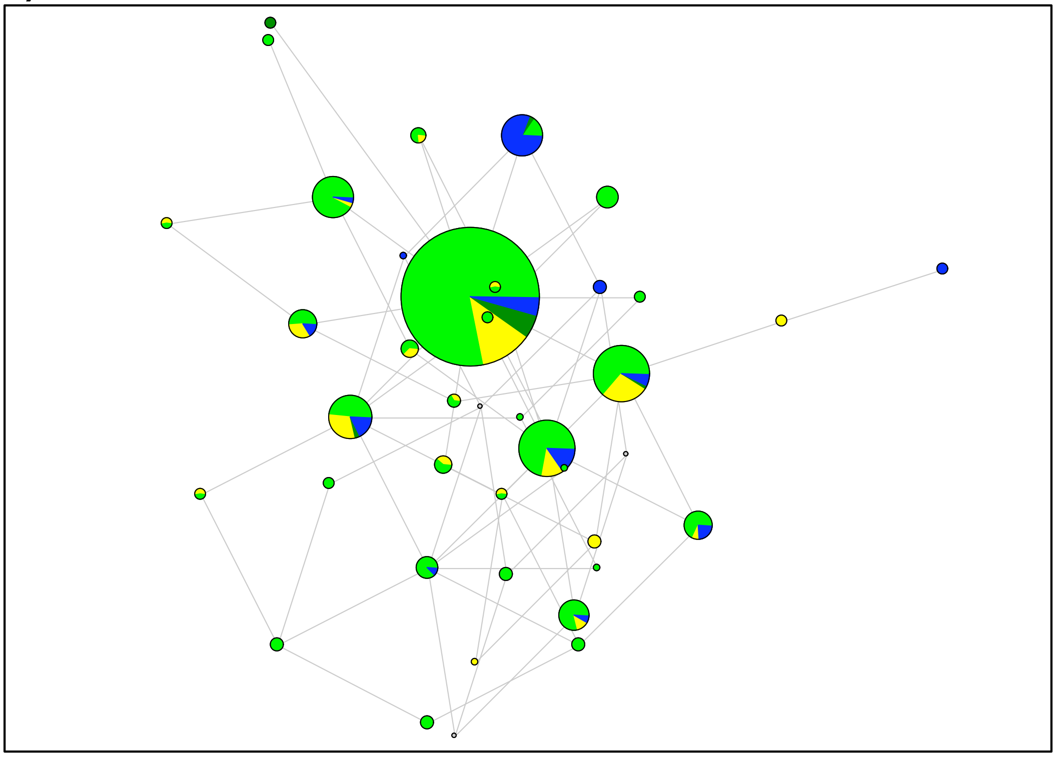

Supplemental Figure S4: Median joining network based on mtDNA HVS-1 haplotypes for all Cross River region samples excluding singletons.

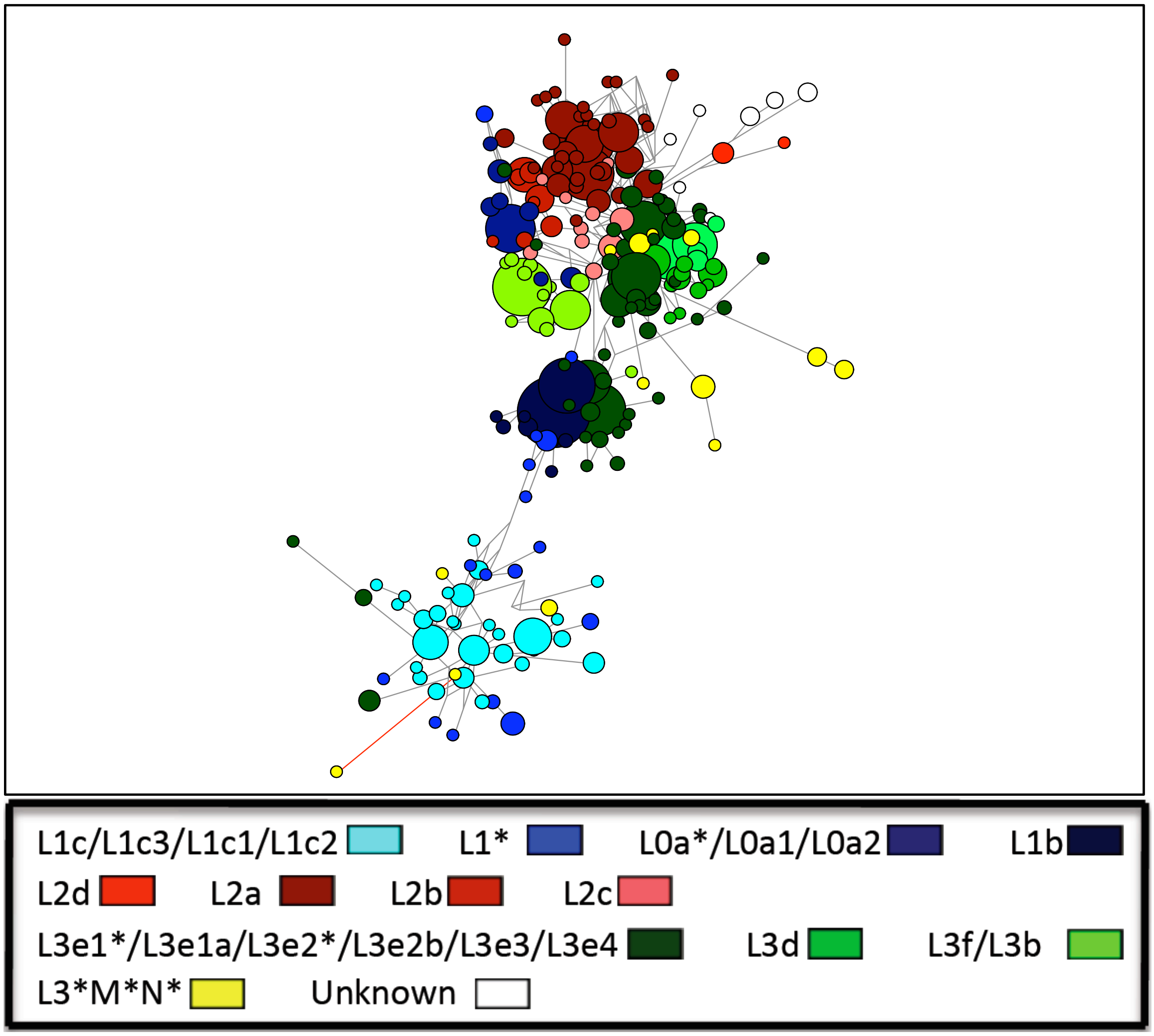

Supplemental Figure S5: Genealogical relationships of UEP markers used to define NRY haplogroups.

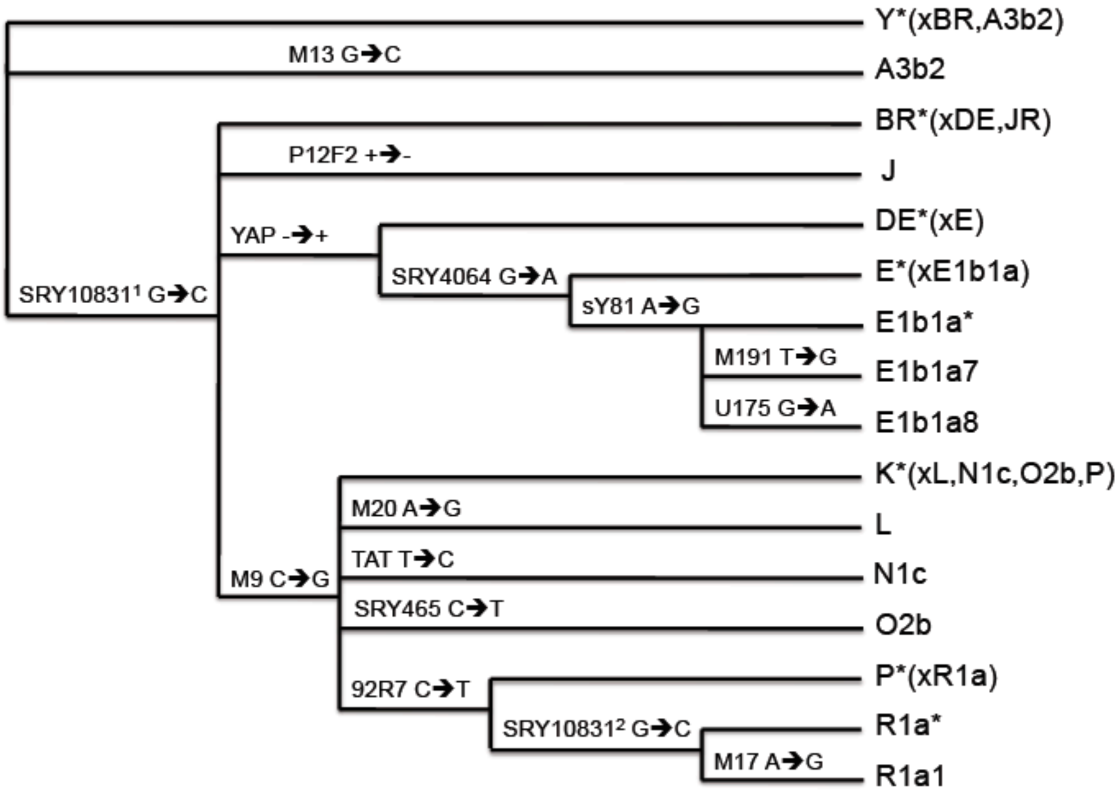

Supplemental Figure S6: Average number of generations significant population pairwise differences (P<0.05) persist as assessed by ETPD across a range of migration rates.

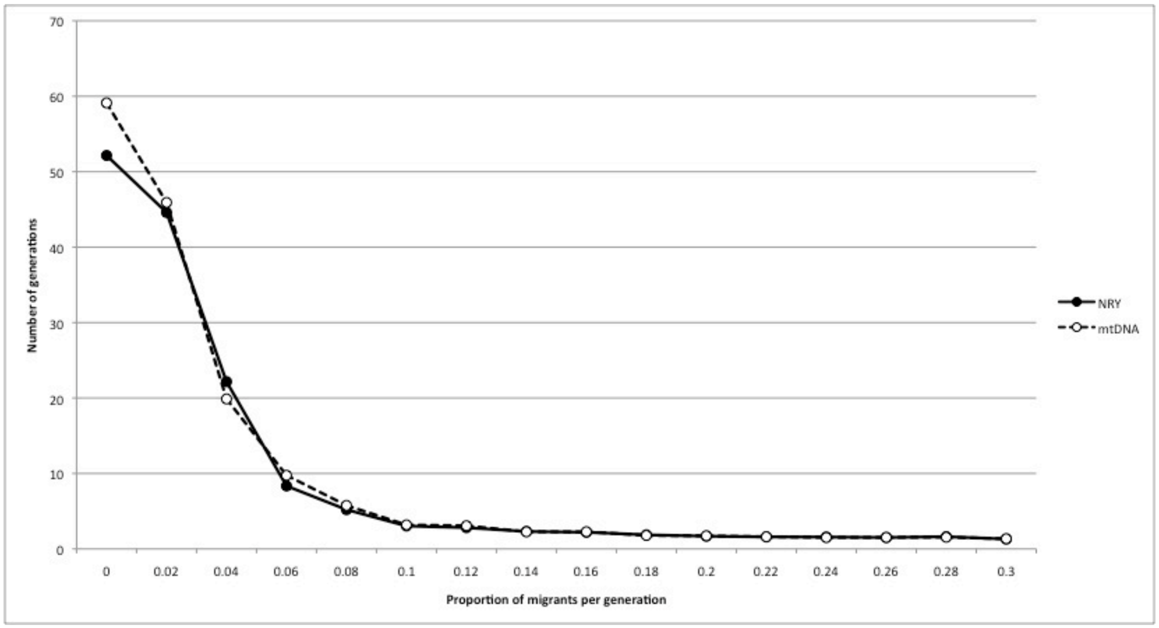

Supplemental Figure S7: Average number of generations significant population pairwise differences (P<0.05) persist as assessed by ETPD across a range of migration rates with varying sample sizes.

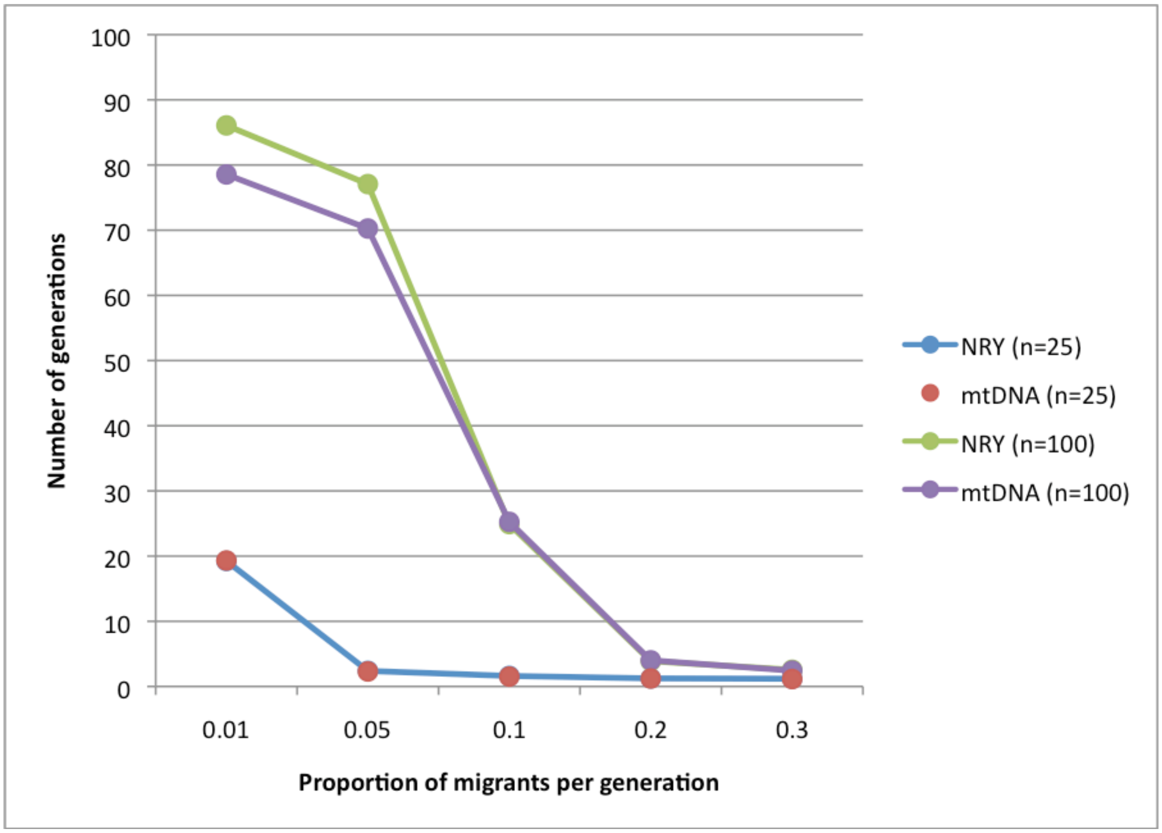

Supplemental Figure S8: Average AMOVA based Fixation Indices in a simulated world across a range of migration rates.

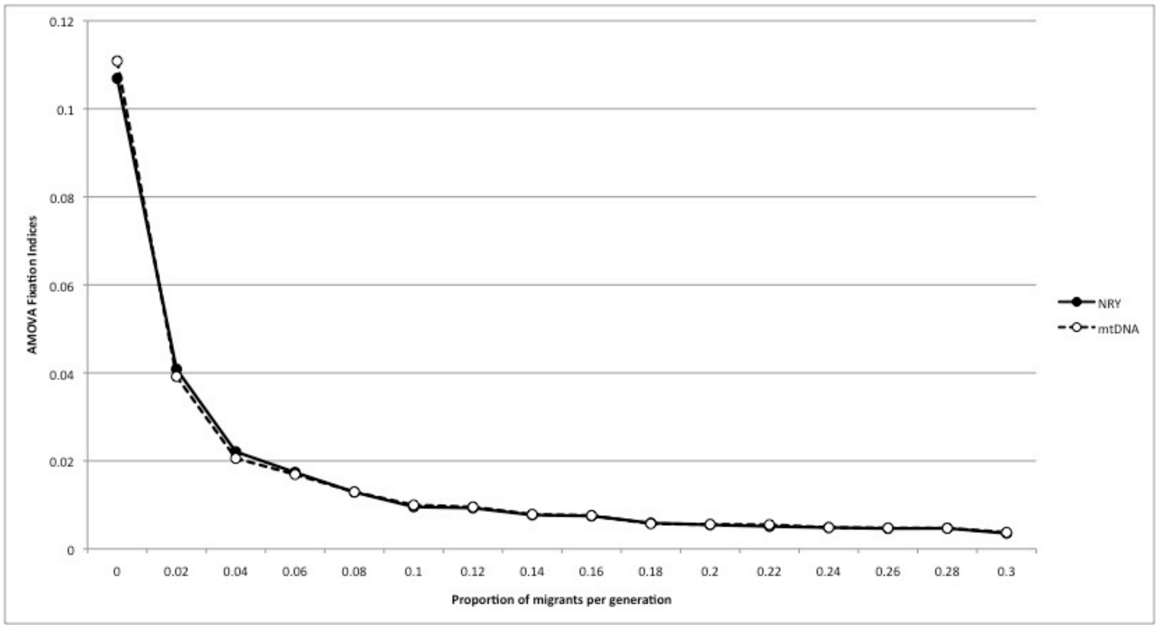

Supplemental Figure S9: Average AMOVA based Fixation Indices in a simulated world across a range of migration rates with varying sample sizes.

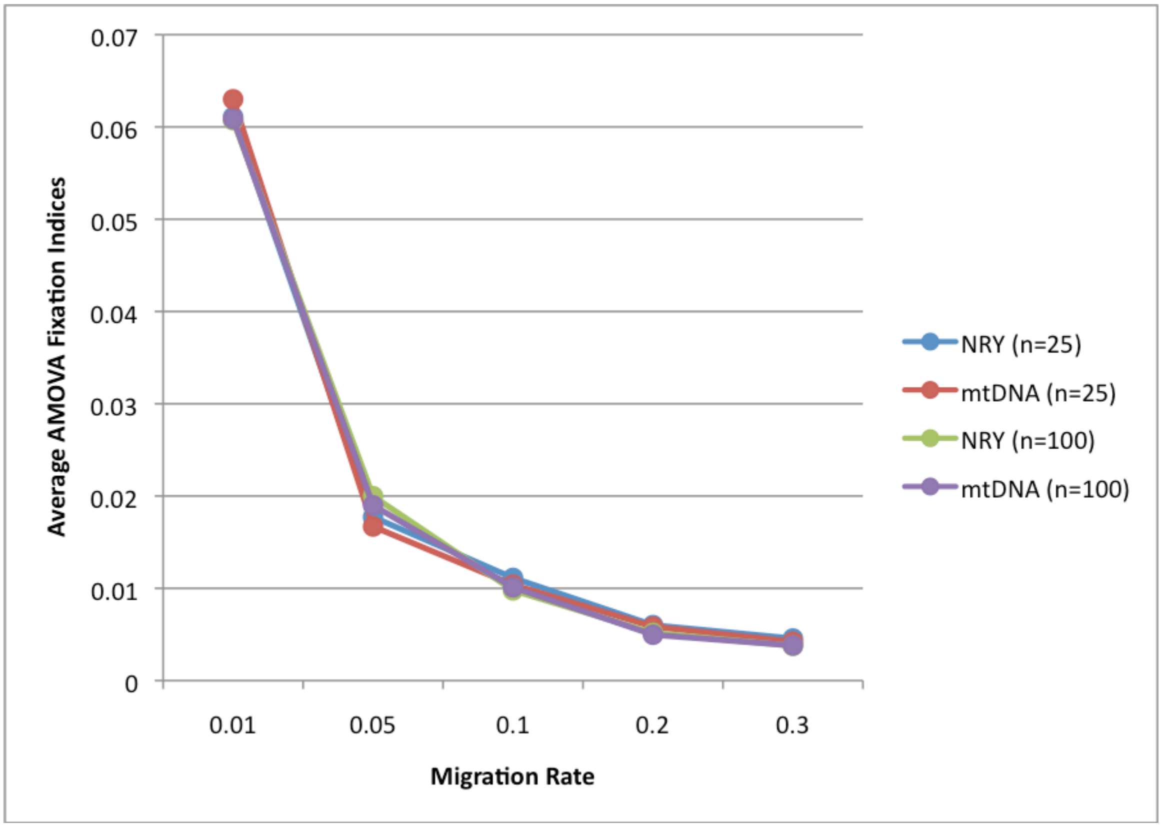

Supplemental Figure S10: Average proportion of significant (P<0.05) pairwise F_{ST} values per generation in simulated world across a range of migration rates.

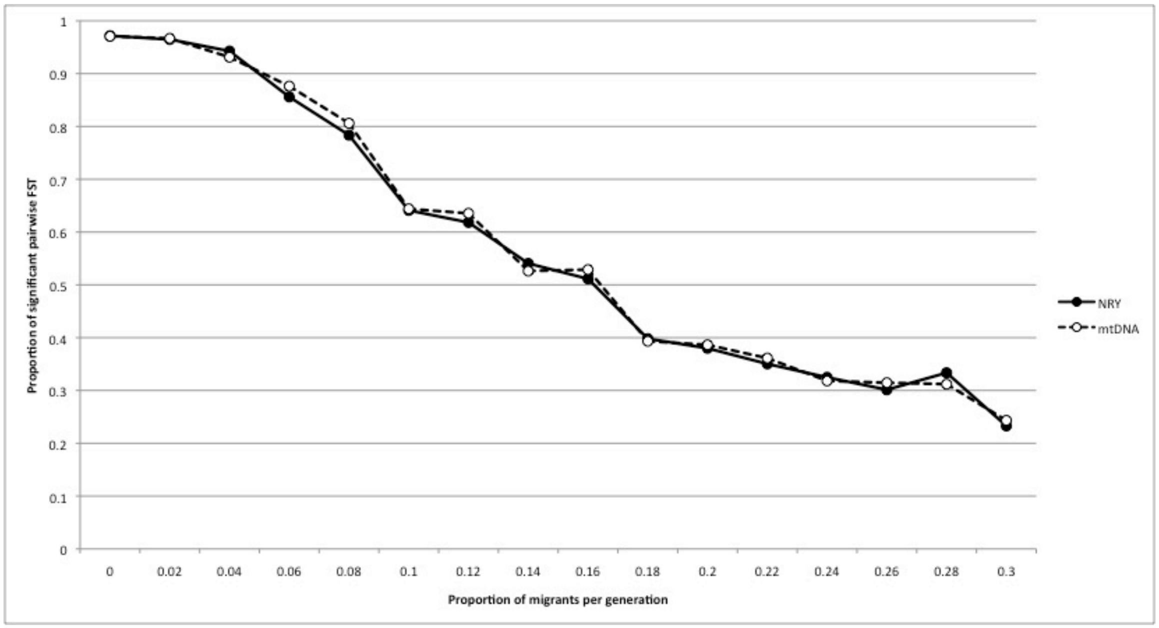

Supplemental Figure S11: Average proportion of significant (P<0.05) pairwise F_{ST} values per generation in simulated world across a range of migration rates with varying sample size.

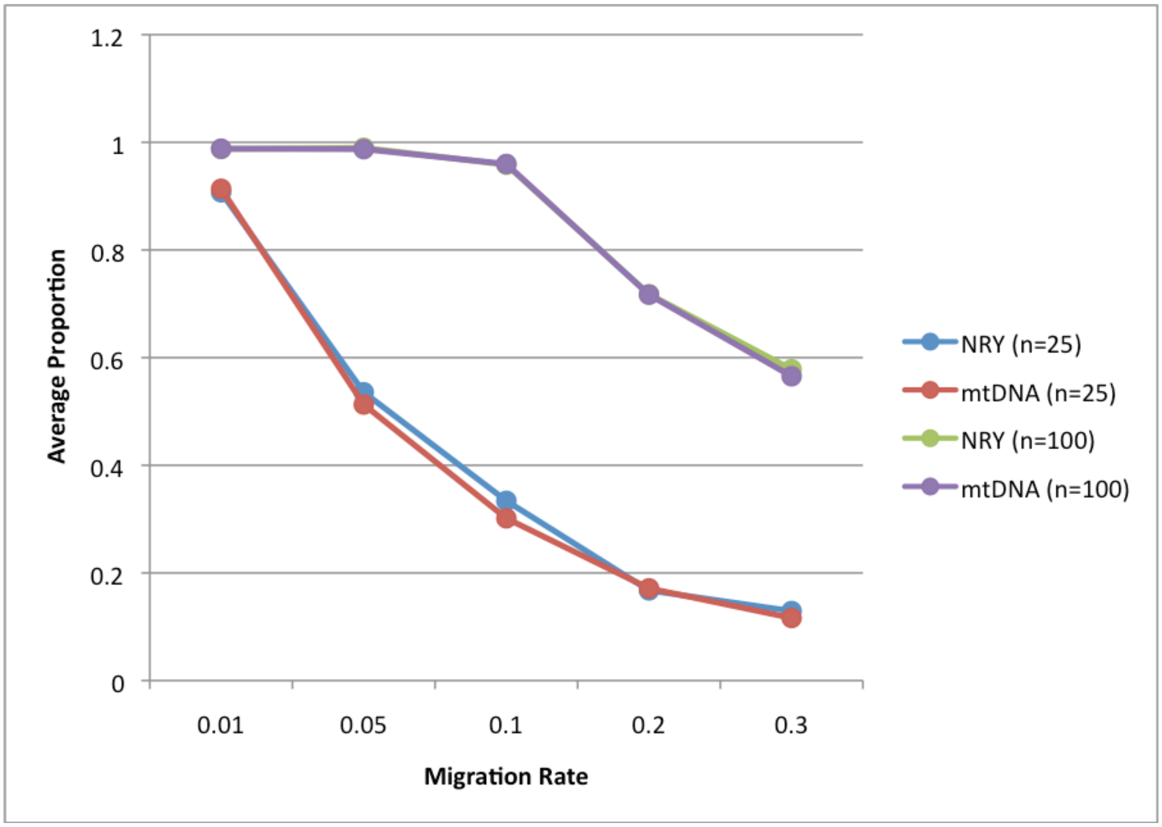

Supplemental Figure S12: Language network based on distance matrix inferred from partial lexicostatistic matrix (Supplemental Table S9).

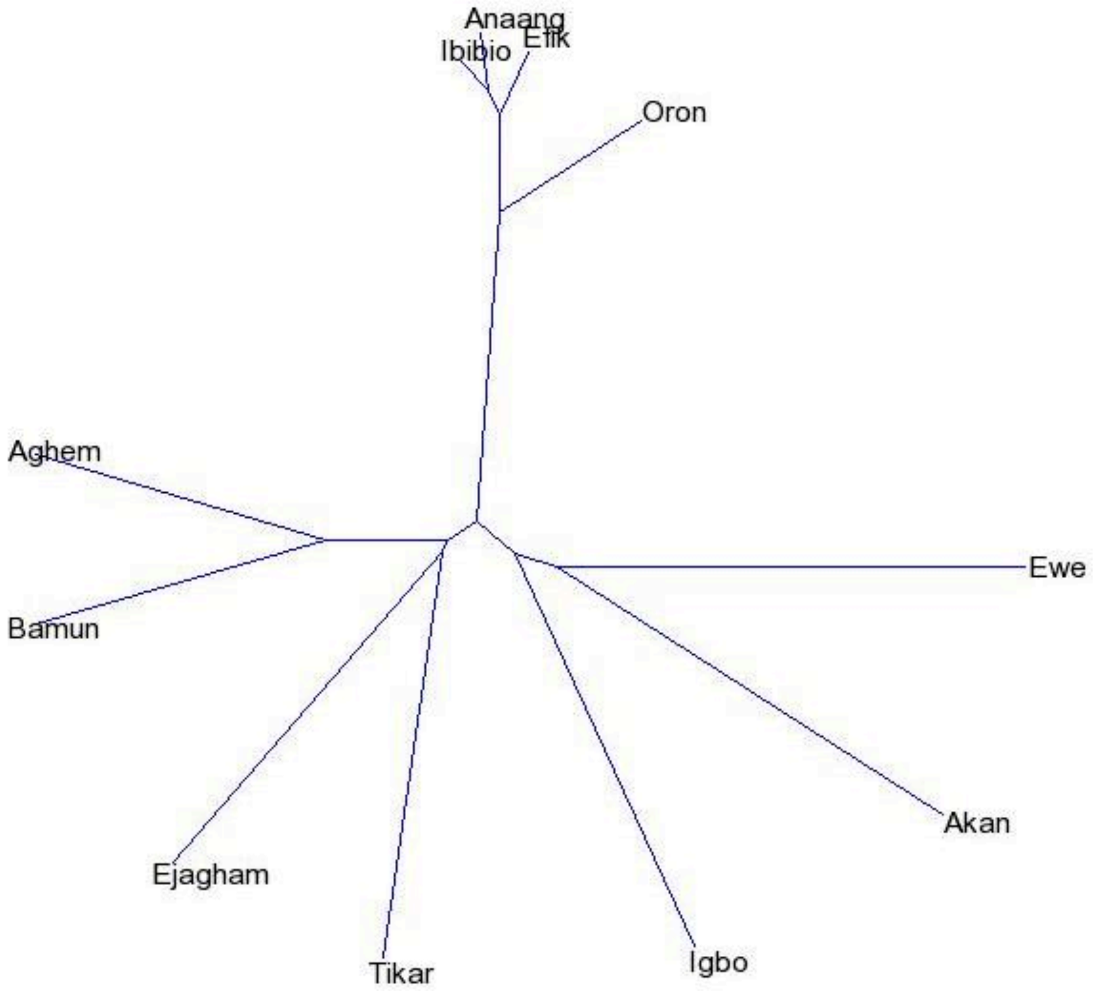

Supplement: Additional file 1 — Supplemental Sections and Figures. A document file containing Supplemental Sections 1-3 and Supplemental Figures S1-S12. [file 1471-2148-10-92-S1.PDF]
